# Prevalence of High Programmed Death-Ligand 1 Expression in Pulmonary Sarcomatoid Carcinoma: A Systematic Review and Meta-Analysis

**DOI:** 10.1016/j.jtocrr.2026.101046

**Published:** 2026-07-10

**Authors:** Yusuke Hamakawa, Aya Shiba, Yohei Noguchi, Hiroyuki Shinohara, Yuko Higashi, Masaharu Aga, Kazuhito Miyazaki, Yuri Taniguchi, Yuki Misumi, Yoko Agemi, Tsuneo Shimokawa, Hiroaki Okamoto, Nobuyuki Horita, Yukiko Nakamura

**Affiliations:** a Department of Respiratory Medicine and Oncology, Yokohama Municipal Citizen’s Hospital, 1-1 Mitsuzawa Nishi Machi, Kanagawa Ku, Yokohama, Kanagawa 221-0855, Japan; b Department of Pulmonology, Graduate School of Medicine, Yokohama City University, Yokohama, Japan

**Keywords:** Pulmonary sarcomatoid carcinoma, Non–small cell lung cancer, Programmed death-ligand 1, Immunotherapy

## Abstract

Pulmonary sarcomatoid carcinoma (PSC) is a rare, aggressive variant of NSCLC associated with emerging evidence of high programmed death-ligand 1 (PD-L1) expression, although its reported prevalence varies widely. We conducted a systematic review and meta-analysis to quantify the pooled prevalence of PD-L1 expression in PSC and identify factors associated with its variability. We searched PubMed, Web of Science, and the Cochrane Library up to November 2025. Studies reporting PD-L1 expression using the tumor proportion score (TPS) in confirmed PSC (≥ 10 patients) were included. A random-effects meta-analysis was performed for TPS thresholds of 50% and 1%. Subgroup analyses and meta-regression were used to explore sources of heterogeneity. This study was registered with PROSPERO (CRD420251243606). Thirty-four studies, encompassing up to 1789 patients, were included. The pooled prevalence of high PD-L1 expression (TPS ≥ 50%) was 57.33%, and PD-L1 positivity (TPS ≥ 1%) was 79.61%. At a TPS of 50% or higher, non-Asian studies reported a numerically higher prevalence than Asian studies, although the difference was not statistically significant. A higher proportion of ever-smokers and a 100% pleomorphic carcinoma composition were each associated with a significantly higher prevalence at a TPS of 1% or higher. In the meta-regression, no covariate reached statistical significance at a TPS of 50% or higher, whereas the proportions of pleomorphic carcinoma, ever-smokers, and median age were significant predictors at a TPS of 1% or higher. PD-L1 expression is remarkably prevalent in PSC, substantially exceeding that in conventional NSCLC. These findings may inform immunotherapy decision-making for advanced PSC.

## Introduction

Pulmonary sarcomatoid carcinoma (PSC) is an exceptionally rare, yet highly aggressive histological variant of NSCLC, representing only 0.1%–0.5% of all primary pulmonary malignancies.^1^^,^^2^ According to the 2021 WHO classification, PSC is categorized into five distinct subtypes: pleomorphic carcinoma, spindle cell carcinoma, giant cell carcinoma, carcinosarcoma, and pulmonary blastoma.^3^ The dual presence of epithelial and sarcomatoid components characterizes these tumors and contributes to their dismal prognosis; patients with advanced-stage tumors experience 5-year survival rates below 5%.^1^^,^^4^ Treatment strategies for PSC have traditionally mirrored those employed for conventional NSCLC; however, clinical outcomes remain profoundly disappointing. Although surgical resection offers the best therapeutic option for localized disease, the 5-year survival rate after complete resection reaches only 11%–24.5%.^4^ Patients with advanced or metastatic PSC derive minimal benefit from platinum-based chemotherapy, with objective response rates of 0%–16.5% and a median overall survival of 4.3–7.9 months.^5^, ^6^, ^7^ Molecular targeted therapies provide a therapeutic benefit in approximately 30% of patients who harbor actionable driver mutations.^8^

The advent of immune checkpoint inhibitors (ICIs) has offered a promising therapeutic alternative for PSC, with multiple studies reporting superior outcomes compared with conventional chemotherapy.^9^^,^^10^ This enhanced immunotherapy responsiveness is attributed in part to the distinctive immunological profile of PSC, most notably the elevated expression of programmed death-ligand 1 (PD-L1). PD-L1 expression, assessed using the tumor proportion score (TPS), is an established predictive biomarker for ICI efficacy in NSCLC; multiple studies have reported that PSC demonstrates markedly higher PD-L1 expression compared with other NSCLC subtypes.^10^, ^11^, ^12^, ^13^

Despite accumulating evidence supporting high PD-L1 expression as a hallmark of PSC, substantial heterogeneity persists across published reports. The reported prevalence of PD-L1 expression varies considerably across studies, ranging from 36.5% to 90.2% for a TPS of 1% or higher and from 21% to 80% for a TPS of 50% or higher,^10^^,^^14^, ^15^, ^16^ likely reflecting differences in patient demographics, geographic region, and histological subtype distribution.

A systematic review and meta-analysis were therefore warranted to synthesize the current evidence on PD-L1 expression patterns in PSC. The present study aimed to (1) quantify the pooled prevalence of high PD-L1 expression (TPS ≥ 50%) and PD-L1 positivity (TPS ≥ 1%) in PSC; (2) identify patient subpopulations with elevated expression rates through subgroup analyses; and (3) explore associations between clinicopathological factors and PD-L1 positivity rates using meta-regression analyses.

## Methods

### Protocol Registration

This systematic review was performed following the Preferred Reporting Items for Systematic Reviews and Meta-Analyses statement. The study protocol was prospectively registered with the International Prospective Register of Systematic Reviews (registration number: CRD420251243606). Because this study was a systematic review and meta-analysis based exclusively on previously published, deidentified, aggregate data and did not involve access to individual patient records, institutional review board approval and informed consent were not required.

### Literature Search Strategy

We systematically searched PubMed, Web of Science, and the Cochrane Library from inception through November 19, 2025. The complete search strategies are provided in Supplementary Table 1. The reference lists of included studies and relevant review articles were manually examined to identify additional eligible publications. Two investigators independently conducted the literature search and screening processes; disagreements were resolved through discussion.

### Eligibility Criteria and Study Design

Original research reporting PD-L1 expression in PSC, including prospective cohorts, retrospective cohorts, cross-sectional studies, and clinical trials, was considered eligible. Conference abstracts with extractable prevalence data were also eligible for inclusion. When multiple publications reported the same or overlapping cohorts, the reports were either combined into a single entry to maximize available data or the most comprehensive report was retained to avoid including duplicate data. We excluded case reports and case series with fewer than 10 patients due to inadequate sample sizes for estimating prevalence. Studies that required PD-L1 positivity for patient enrollment were also excluded, as such designs preclude unbiased prevalence estimation. Only English-language publications were considered.

### Patient Population

Studies enrolling patients with pathologically confirmed PSC were included. All histological subtypes recognized by the WHO classification—pleomorphic carcinoma, spindle cell carcinoma, giant cell carcinoma, carcinosarcoma, and pulmonary blastoma—were eligible.^3^ No restrictions were imposed on disease stage, prior treatment history, performance status, or patient age.

### PD-L1 Evaluation

Studies were required to report PD-L1 protein expression assessed using immunohistochemistry on tumor specimens using the TPS, defined as the percentage of viable tumor cells showing partial or complete membranous staining, reported at the 1% cutoff, the 50% cutoff, or both. To ensure consistency, studies that used nonmembranous (cytoplasmic) scoring, intensity-based or quantitative-fluorescence scoring, H-scores, combined positive scores, or nonstandard positivity thresholds (e.g., a ≥ 25% cutoff) were considered incompatible with this definition and were excluded. When the antibody clone was unspecified, a study was retained only if PD-L1 expression was explicitly reported as TPS at the 1% or 50% cutoffs. All commercially available antibody clones were otherwise acceptable; studies using noncommercial antibodies or evaluating PD-L1 expression exclusively in tumor-infiltrating immune cells were excluded. The specific clones used in each study are listed in the footnote of Table 1.

**Table 1:** Characteristics of the 34 Studies Included in the Meta-Analysis

Study	Country	Design	N (total)	n (PD-L1)	Median Age	Men (%)	Smoker (%)	PC (%)	PD-L1 ≥1% n (%)	PD-L1 ≥50% n (%)
Alì et al.^17^	Italy	Retrospective	44	44	—	—	—	47.7	37 (84.1)	21 (47.7)
Amrane et al.^18^	France	Retrospective	161	70	66	70.2	86.2	—	54 (77.1)	48 (68.6)
Chan et al.^19^	China	Retrospective	21	21	—	—	—	—	16 (76.2)	11 (52.4)
Cheng et al.^20^ and Wang et al.^21^	US	Retrospective	41	36	73	48.8	92.1	97.3	27 (75.0)	21 (58.3)
Domblides et al.^9^	France	Retrospective	37	19	63	73	94.6	—	18 (94.7)	13 (68.4)
Du et al.^22^	China	Phase II	38	34	—	92.1	86.8	—	32 (94.1)	22 (64.7)
Eldessouki et al.^23^	US	Retrospective	53	53	—	64.2	—	—	42 (79.2)	—
Hazama et al.^24^	Japan	Retrospective	124	116	69	78.2	85.5	74.2	105 (90.5)	81 (69.8)
Hong et al.^25^	USA	Retrospective	178	132	—	51.1	86	—	117 (88.6)	99 (75.0)
Imanishi et al.^16^	Japan	Retrospective	26	26	68	80.8	84.6	100	20 (76.9)	—
Kaira et al.^26^	Japan	Retrospective	99	99	69	74.7	80.8	100	84 (84.8)	21 (21.2)
Kojima et al.^27^	Japan	Cross-sectional	11	11	—	—	—	100	11 (100.0)	9 (81.8)
Kwon et al.^28^	Korea	Retrospective	31	31	—	80.6	74	90.3	—	19 (61.3)
Lee et al.^10^	Korea	Retrospective	175	122	—	—	—	100	106 (86.9)	91 (74.6)
Li et al.^29^	Australia	Retrospective	28	28	73	78.6	89.3	—	26 (92.9)	23 (82.1)
Ma et al.^30^	China	Retrospective	32	32	—	90.6	62.5	75	19 (59.4)	11 (34.4)
Nagase et al.^31^	Japan	Retrospective	71	71	71	83	90	100	62 (87.3)	22 (31.0)
Naito et al.^32^	Japan	Retrospective	35	35	65	71.4	82.9	100	32 (91.4)	21 (60.0)
Ng Kee Kwong et al.^33^	UK	Retrospective	21	21	—	—	—	100	21 (100.0)	19 (90.5)
Noguchi et al.^34^	Japan	Retrospective	46	46	70.5	89.1	87	100	37 (80.4)	15 (32.6)
Schenk et al.^35^	USA	Retrospective	30	27	69	60	90.3	—	21 (77.8)	—
Shishido et al.^36^	Japan	Retrospective	29	29	70	69	75.9	100	22 (75.9)	14 (48.3)
Sikora et al.^37^	Poland	Retrospective	107	107	—	—	—	—	68 (63.6)	—
Sim et al.^38^	Korea	Retrospective	26	13	69.5	88.5	92.3	—	12 (92.3)	8 (61.5)
Stephan-Falkenau et al.^39^	Germany	Retrospective	179	179	—	58.7	85.2	64.8	147 (82.1)	106 (59.2)
Tancoš et al.^40^	Slovakia	Retrospective	32	32	—	—	—	100	26 (81.3)	19 (59.4)
Wei et al.^41^	China	Retrospective	33	16	61	81.8	66.7	—	10 (62.5)	—
Wu et al.^42^	China	Retrospective	24	24	63.5	83.3	79.2	66.7	21 (87.5)	17 (70.8)
Wu et al.^43^	China	Retrospective	60	30	65	81.7	76.7	53.5	24 (80.0)	18 (60.0)
Yang et al.^14^	China	Retrospective	148	148	62.5	79.1	70.3	—	54 (36.5)	—
Yvorel et al.^44^	France	Retrospective	36	36	—	80.6	—	86.1	27 (75.0)	16 (44.4)
Zhang et al.^45^	China	Retrospective	38	38	61	78.9	63.9	—	21 (55.3)	—
Zhou et al.^46^	China	Retrospective	58	58	63	86.2	50	0	26 (44.8)	14 (24.1)
Zhou et al.^47^	China	Retrospective	42	36	64	78.6	45.2	—	31 (86.1)	19 (52.8)

N (total), total number of patients in the study cohort; n (PD-L1), number of patients with PD-L1 expression evaluated; PC, pleomorphic carcinoma.

PD-L1 was assessed using clone 22C3 unless otherwise noted. Alternative clones: Cheng et al.^20^/Wang et al.^21^, SP142; Nagase et al.^31^, Noguchi et al. ,^34^ and Yang et al.,^14^ 28-8; Schenk et al.^35^, Shishido et al.^36^, Zhou et al.^47^, Yvorel et al.^44^, and Zhang et al.^45^, E1L3N; Stephan-Falkenau et al.^39^, 73-10. Antibody clone was not specified in Wu et al.^42^

—, data not reported or not applicable. Abbreviations: PD-L1, programmed death-ligand 1; PC, pleomorphic carcinoma; TPS, tumor proportion score.

Studies that reported PD-L1 in PSC but were excluded because the antibody or scoring method was incompatible with the tumor proportion score definition are listed in the Supplementary Appendix.

### Risk of Bias Assessment

The methodological quality of the included studies was evaluated using the Joanna Briggs Institute Critical Appraisal Checklist for Studies Reporting Prevalence Data,^48^ which appraises nine domains of study quality.

### Outcome Measures

The primary outcome was the pooled prevalence of high PD-L1 expression, defined asa TPS of 50% or higher, as this threshold has served as an inclusion criterion for immunotherapy trials and has reported an association with favorable treatment outcomes in PSC.^10^^,^^49^ The secondary outcome was the pooled prevalence of PD-L1 positivity, defined as a TPS of 1% or higher.

### Data Collection

Using a standardized data extraction form, two reviewers independently collected the following information from each eligible study: the first author, publication year, country, study design, total number of patients, median age, sex distribution, smoking history, distribution of histological subtypes, and number of patients with PD-L1 expression at a TPS of 50% or higher and a TPS of 1% or higher. Any discrepancies between the reviewers were resolved through consensus or consultation with a third reviewer.

### Subgroup Analyses

To investigate the potential sources of heterogeneity, we performed preplanned subgroup analyses stratified by geographic region (Asian versus non-Asian populations), median age (using the median value as a cutoff), proportion of male patients (using the median value as a cutoff), proportion of ever-smokers (using the median value as a cutoff), and proportion of pleomorphic carcinoma cases (using the median value as a cutoff). Because the cutoffs were derived from study-level medians rather than clinically established thresholds, and the covariates represent aggregate study-level characteristics, these subgroup analyses were regarded as exploratory and hypothesis-generating, and their full results are presented in the Supplementary Material.

### Statistical Analysis

We conducted a random-effects meta-analysis to pool prevalence estimates, employing the generic inverse variance method with the DerSimonian-Laird estimator for between-study variance, implemented using the “meta” package in R software, version 4.3.3 (R Foundation for Statistical Computing, Vienna, Austria). Raw proportions (expressed as percentages) were used as point estimates, with standard errors calculated using the Agresti-Coull adjustment. Between-study heterogeneity was evaluated using the I^2^ statistic and Cochran’s Q test (*p* < 0.10); I^2^ values were categorized as low (< 25%), moderate (25%–75%), or high (> 75%). Differences between subgroups were assessed using the chi-squared test, with *p* less than 0.05 considered statistically significant.

Meta-regression analyses were performed using the restricted maximum likelihood method with the “metafor” package in R software, with logit transformation applied before regression modeling. Five covariates were examined in univariate meta-regression: geographic region (Asian versus non-Asian), median age, proportion of men, proportion of ever-smokers, and proportion of pleomorphic carcinoma. The R^2^ statistic was calculated to quantify the proportion of between-study heterogeneity explained by each covariate.

Publication bias was assessed through visual inspection of contour-enhanced funnel plot asymmetry and the Egger’s regression test, both of which were based on logit-transformed prevalence estimates. Duval and Tweedie’s trim-and-fill method was applied to estimate the number of potentially missing studies and to calculate adjusted pooled prevalence estimates. A leave-one-out sensitivity analysis was conducted by sequentially omitting each study and recalculating the pooled prevalence to evaluate the influence of individual studies on the overall estimates. All tests for meta-regression and publication bias were two-sided, with *p* less than 0.05 considered statistically significant.

## Results

### Study Selection

**Figure 1:**
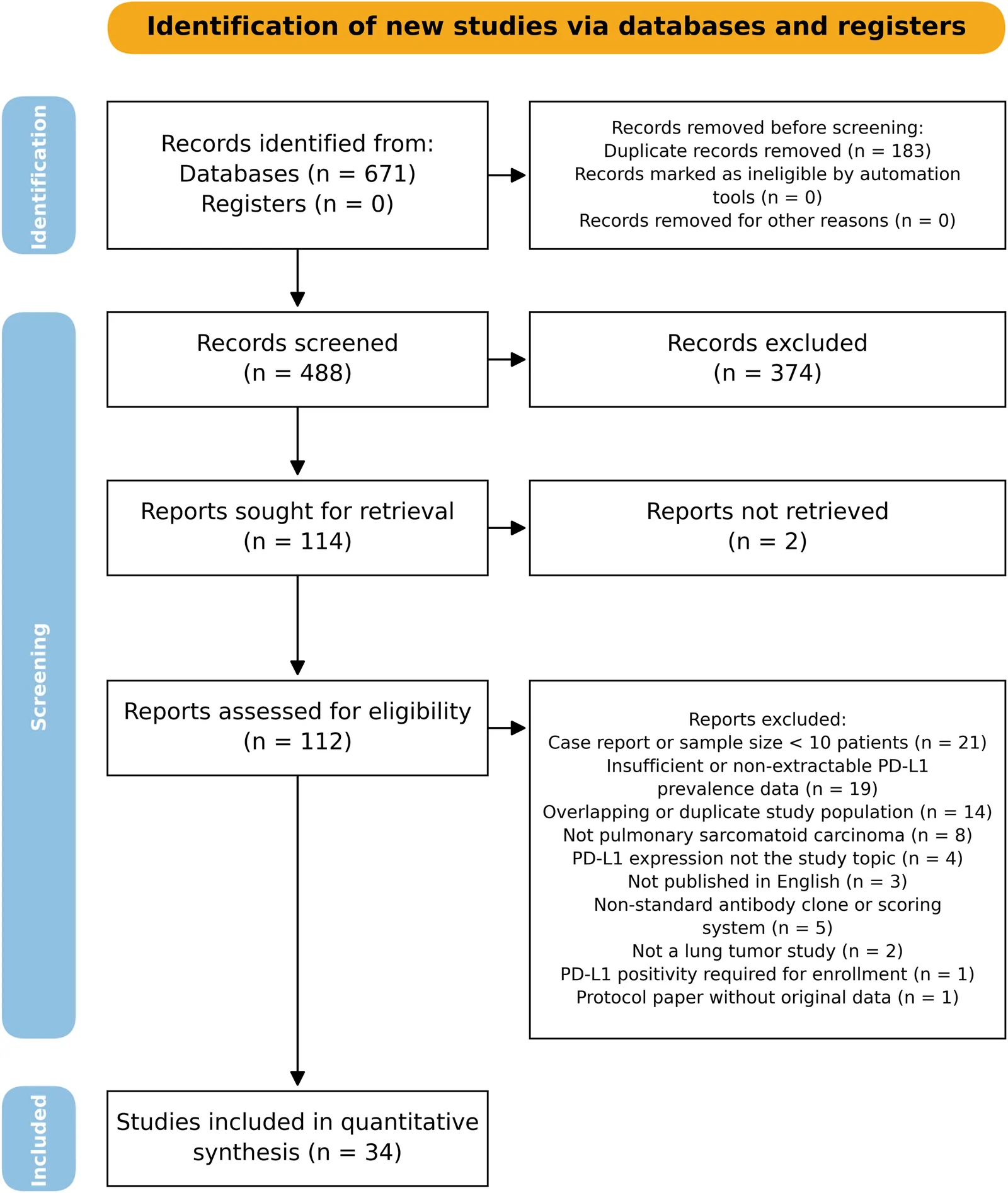
PRISMA flow diagram of the study selection process. PD-L1, programmed death-ligand 1; PRISMA, Preferred Reporting Items for Systematic Reviews and Meta-Analyses.

The database search identified 671 records: 373 from PubMed, 281 from Web of Science, and 17 from Cochrane CENTRAL. After removing 183 duplicates, 488 records underwent title and abstract screening, and 374 were excluded. Of the 114 reports remaining for full-text retrieval, two were not retrieved. A total of 112 reports were assessed for eligibility, and 78 were excluded (reasons detailed in Fig 1). Ultimately, 34 studies were included in the quantitative synthesis (Figure 1). Among these, one entry (Cheng et al.^20^ and Wang et al.^21^) combined two reports describing the same cohort to maximize available data, and one conference abstract (Lee, 2017^50^) that reported the same cohort as a subsequent full-text article^38^ was excluded as a duplicate publication. During full-text assessment, three further reports that might at first seem eligible were excluded; because each is closely related to PSC, the reason for excluding each is given individually: Wu, 2024^51^ was removed as a duplicate of Du et al.,^22^ as both describe the same Shanghai Pulmonary Hospital phase II cohort (ChiCTR2000031478), and the more complete report^22^ was retained; Chang, 2022^52^ was excluded because it comprised non–small cell carcinoma with spindle or giant cell features (NSCLCsg) diagnosed predominantly on small biopsies, which cannot be reliably equated with histologically confirmed PSC; and Ağaçkıran, 2021^53^ was excluded from the meta-analysis (both the TPS of 1% or higher and TPS of 50% or higher analyses) owing to a nonstandard PD-L1 scoring approach (membranous or cytoplasmic staining with a ≥ 25% positivity cutoff) that is incompatible with the 22C3-based TPS definition at either threshold (see PD-L1 evaluation in Methods). The characteristics of the included studies are summarized in Table 1.

### Risk of Bias Assessment

The methodological quality of the 34 included studies was generally high (Supplementary Table 2). The median JBI score was 8.5 out of 9 (range, 4–9), with 32 studies (94.1%) scoring 7 or above. Two studies (Eldessouki et al.^23^; Sikora^37^; and Langfort^23^) scored 4 out of 9, primarily because of unclear sampling methods, insufficient descriptions of study subjects, and unclear reporting of coverage and response rates. These two studies were retained in the primary analysis, and their potential influence was evaluated through a leave-one-out sensitivity analysis.

### Pooled Prevalence of PD-L1 Expression

**Figure 2:**
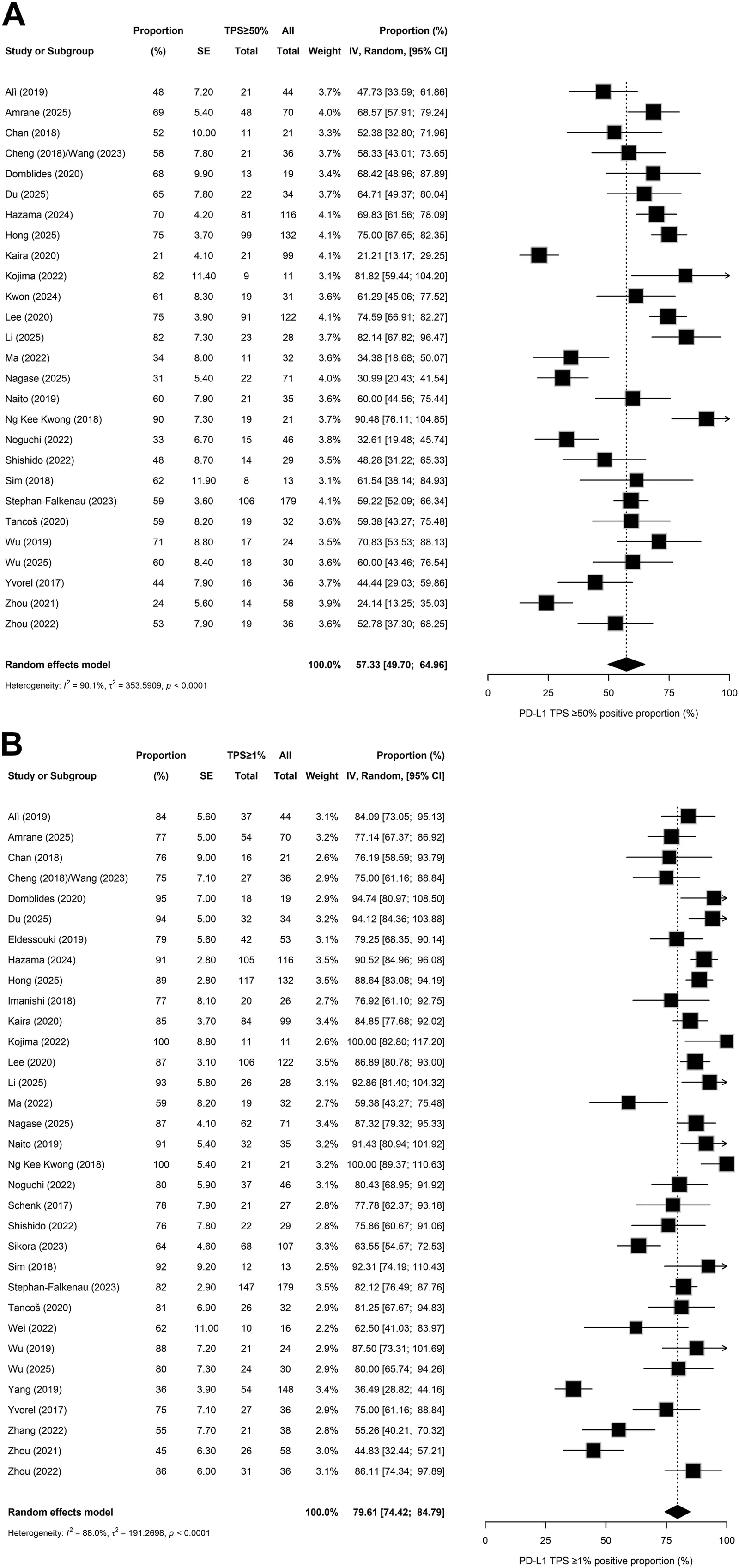
Forest plots of the pooled prevalence of PD-L1 expression in pulmonary sarcomatoid carcinoma. (*A*) High PD-L1 expression at the TPS ≥ 50% threshold (k = 27); (*B*) PD-L1 positivity at the TPS ≥ 1% threshold (k = 33). PD-L1, programmed death-ligand 1; TPS, tumor proportion score.

For the TPS of 50% or higher threshold, 27 studies encompassing 1405 patients were included in the meta-analysis. The pooled prevalence of high PD-L1 expression was 57.33% (95% CI: 49.70%–64.96%), with substantial between-study heterogeneity (I^2^ = 90.1%; τ^2^ = 353.59; *p* < 0.0001) (Fig 2*A*). The prevalence in individual studies ranged from 21.2% to 90.5%.

For the TPS of 1% or higher threshold, 33 studies encompassing 1789 patients were included. The pooled prevalence of PD-L1 positivity was 79.61% (95% CI: 74.42%–84.79%), with substantial heterogeneity (I^2^ = 88.0%; τ^2^ = 191.27; *p* < 0.0001) (Fig 2*B*). The individual study prevalence ranged from 36.5% to 100%.

### Subgroup Analyses

**Supplementary Figure 1:**
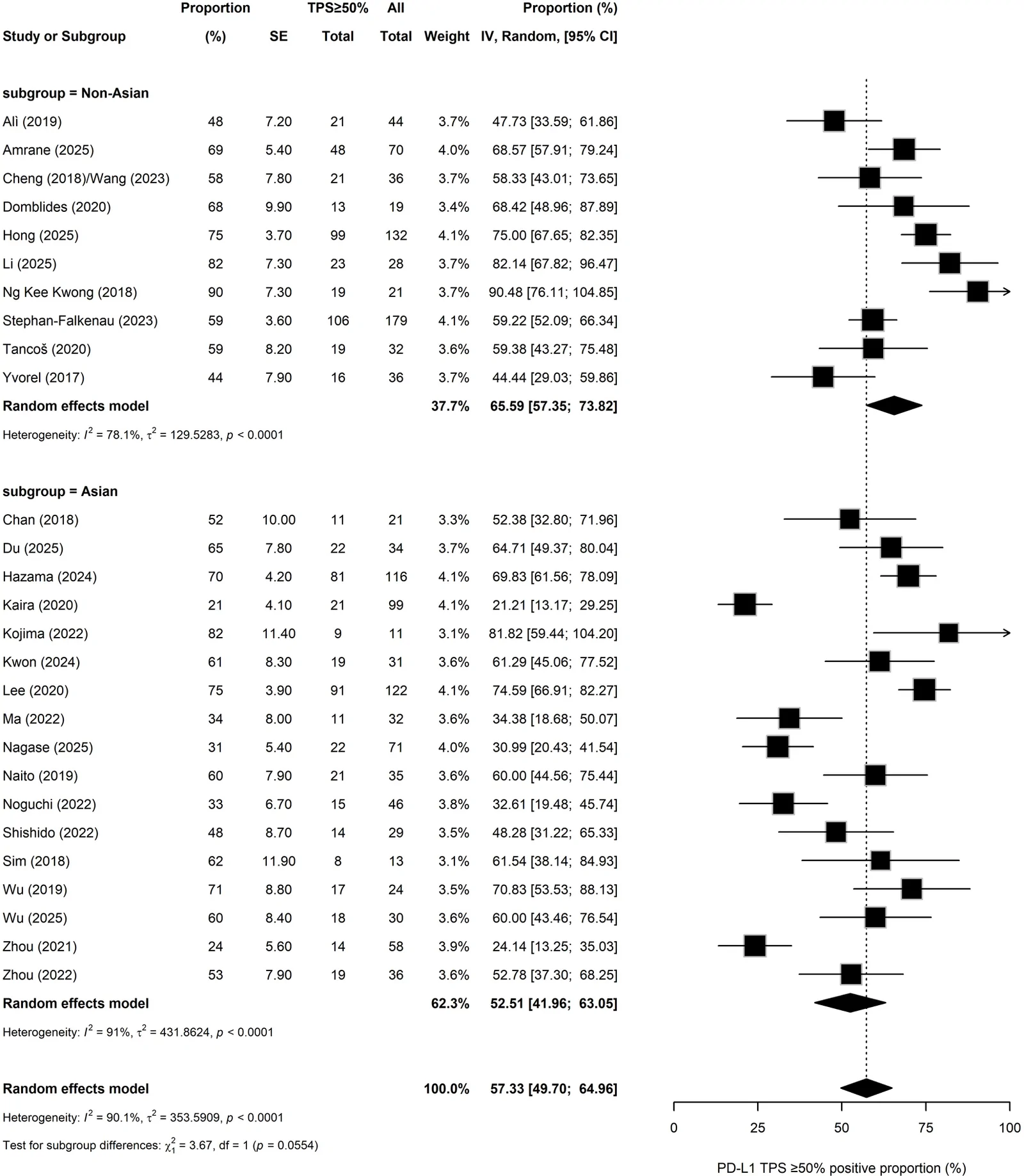


**Supplementary Figure 2:**
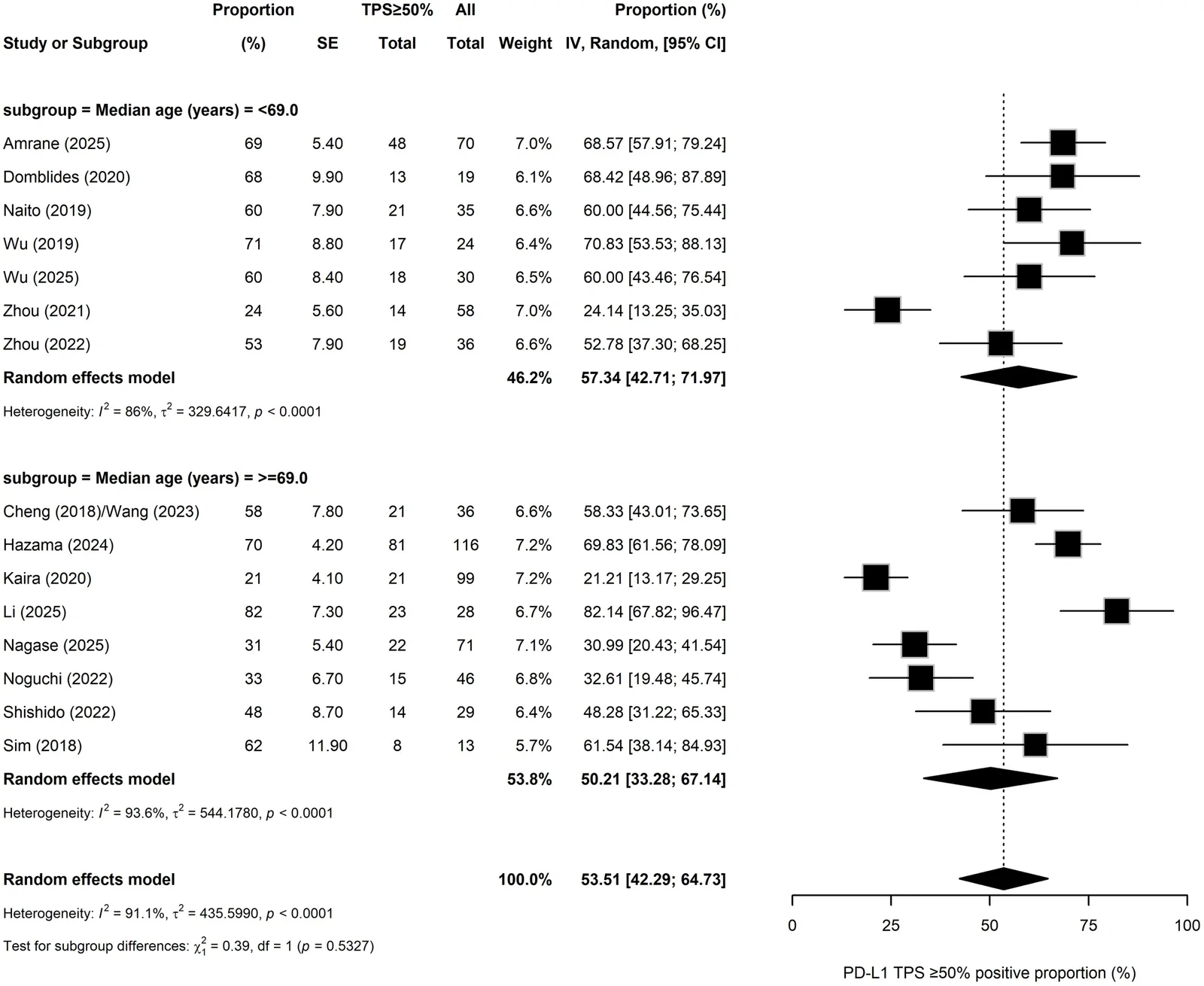


**Supplementary Figure 3:**
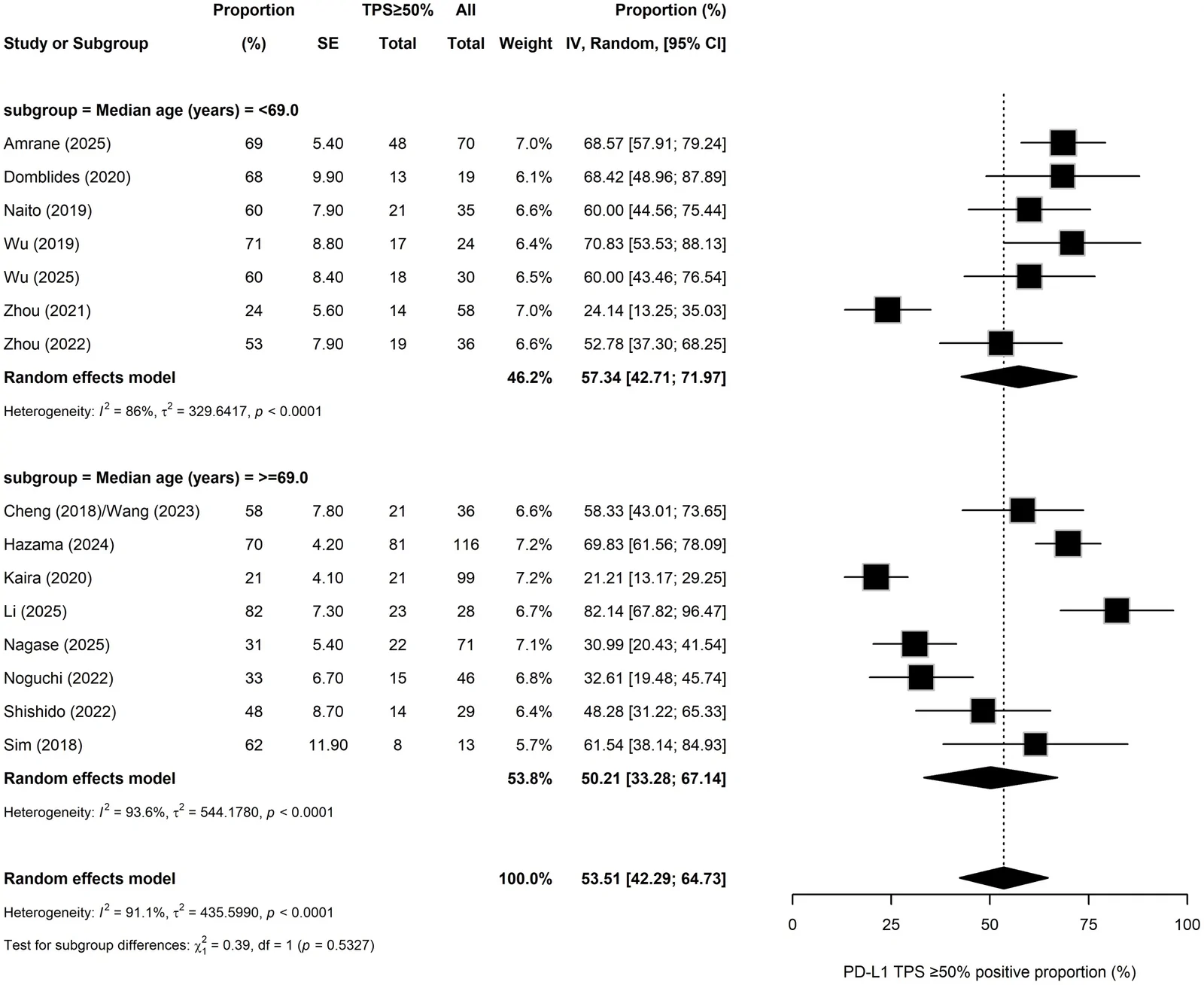


**Supplementary Figure 4:**
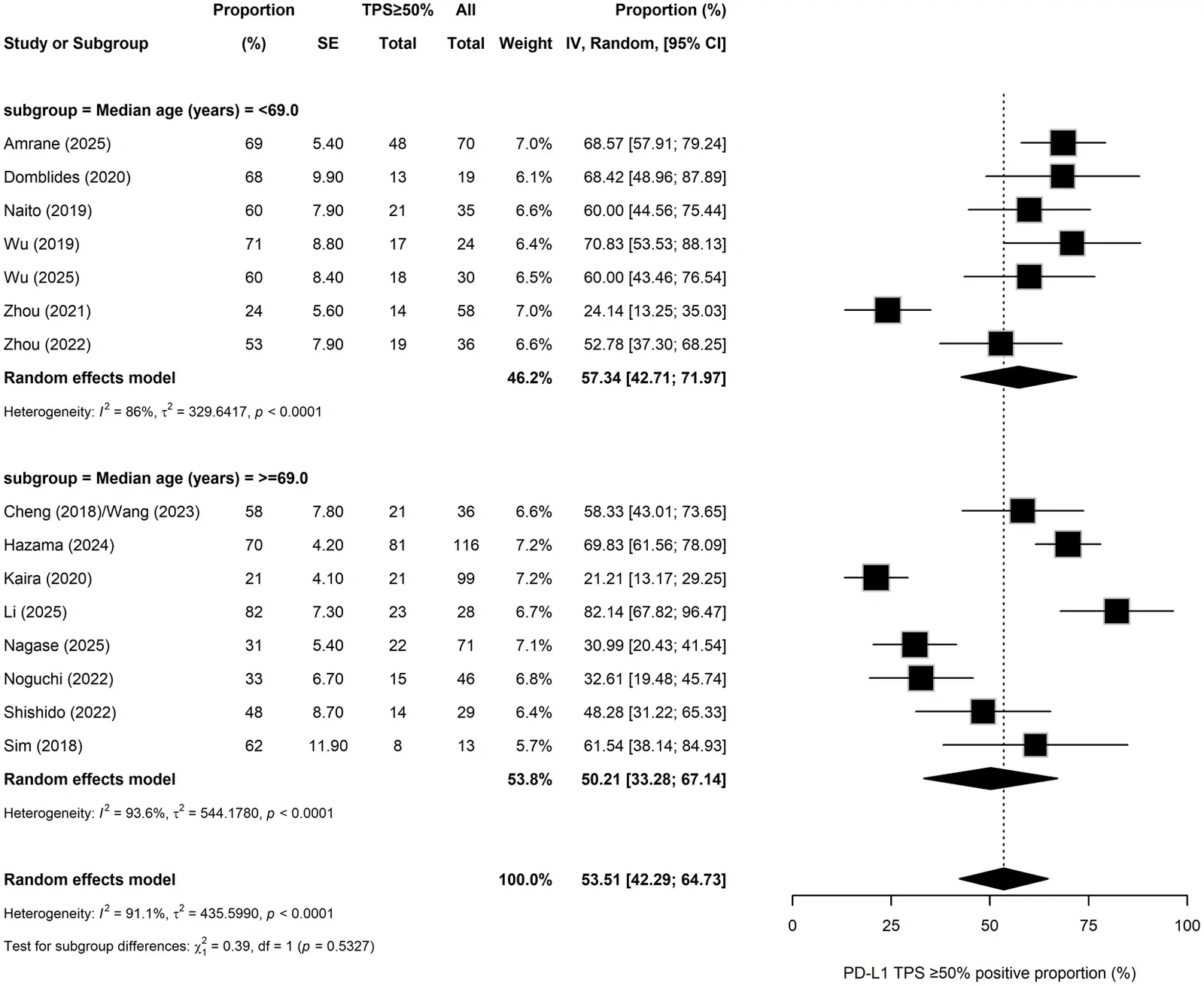


**Supplementary Figure 5:**
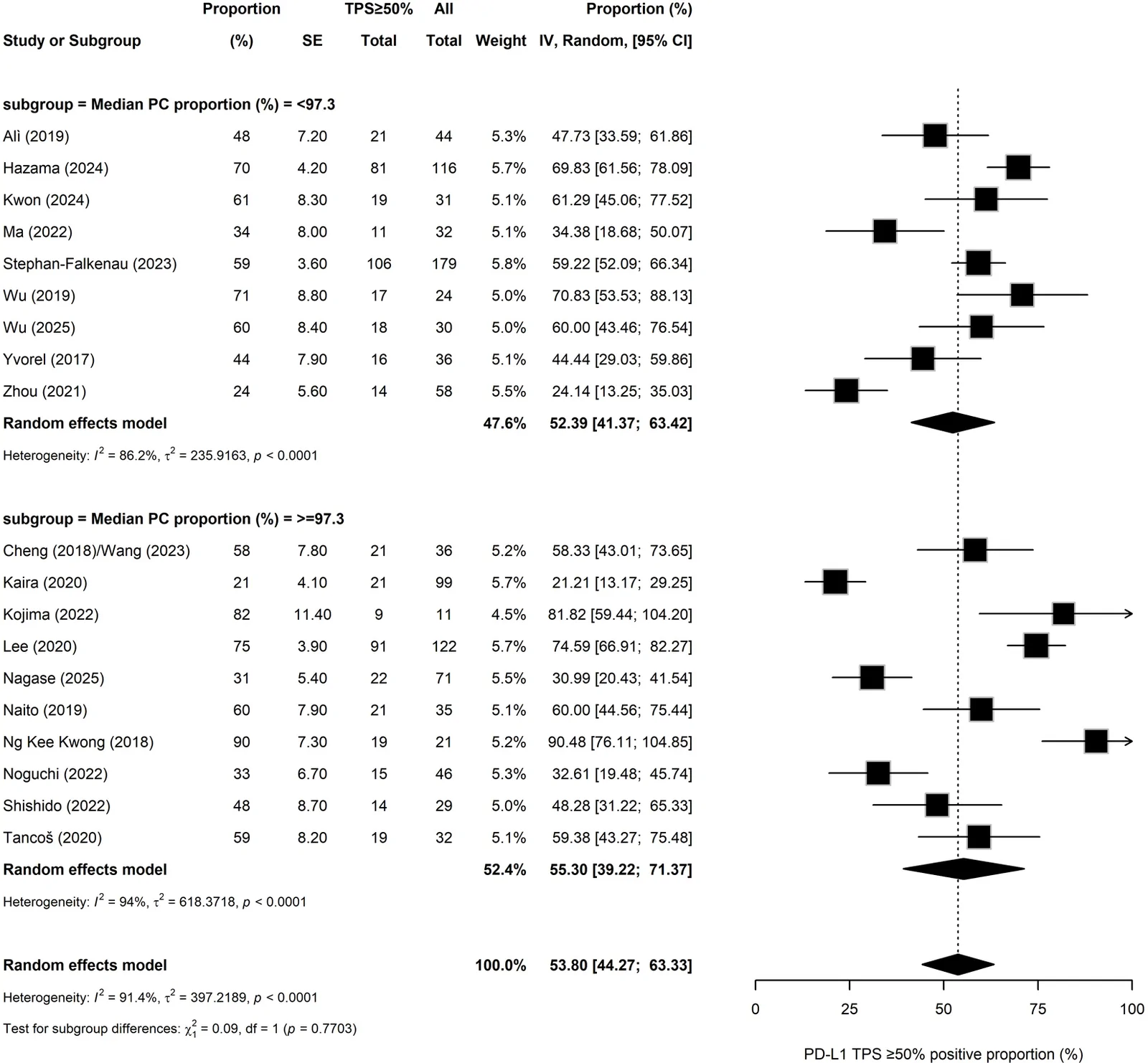


**Supplementary Figure 6:**
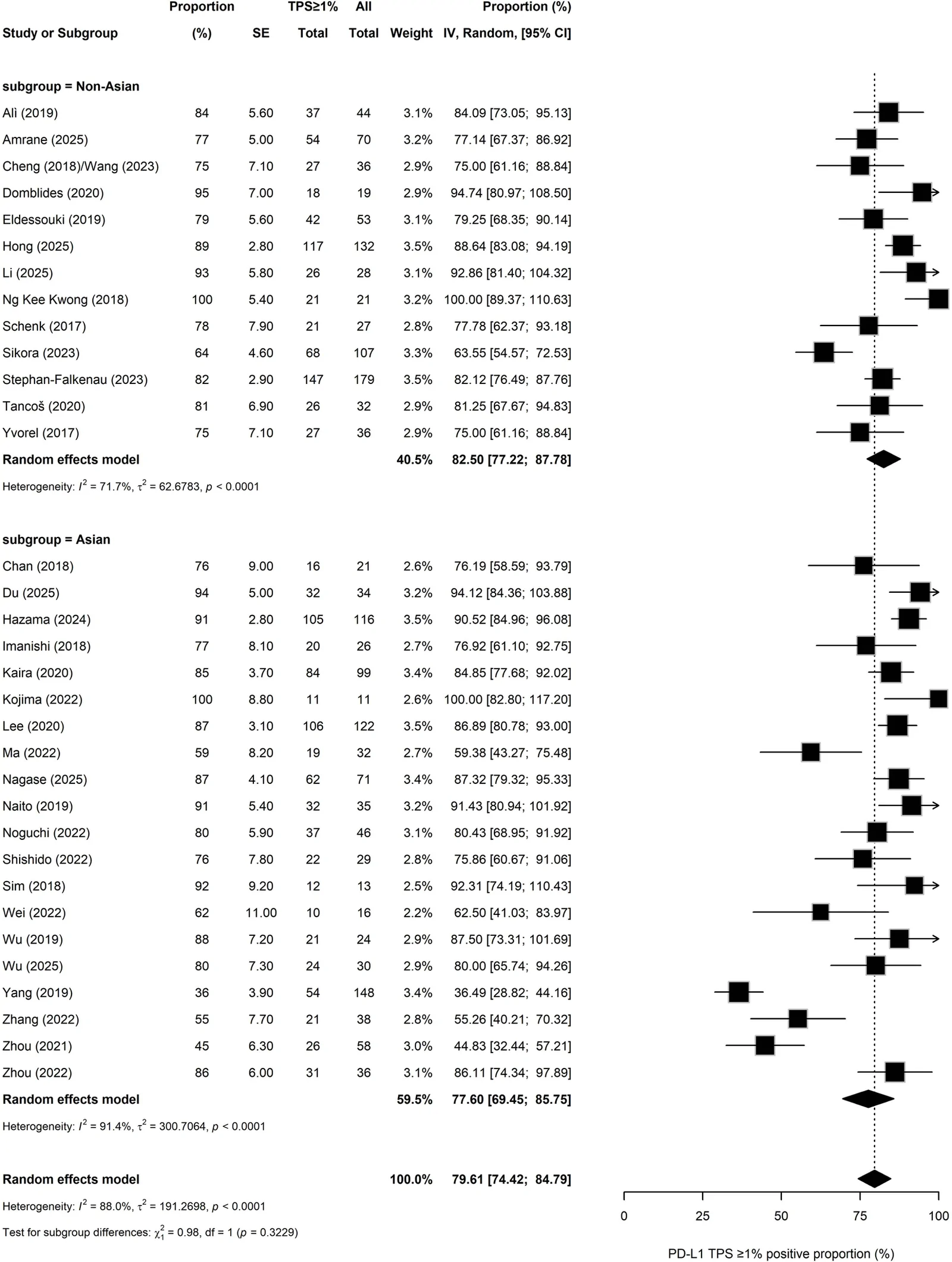


**Supplementary Figure 7:**
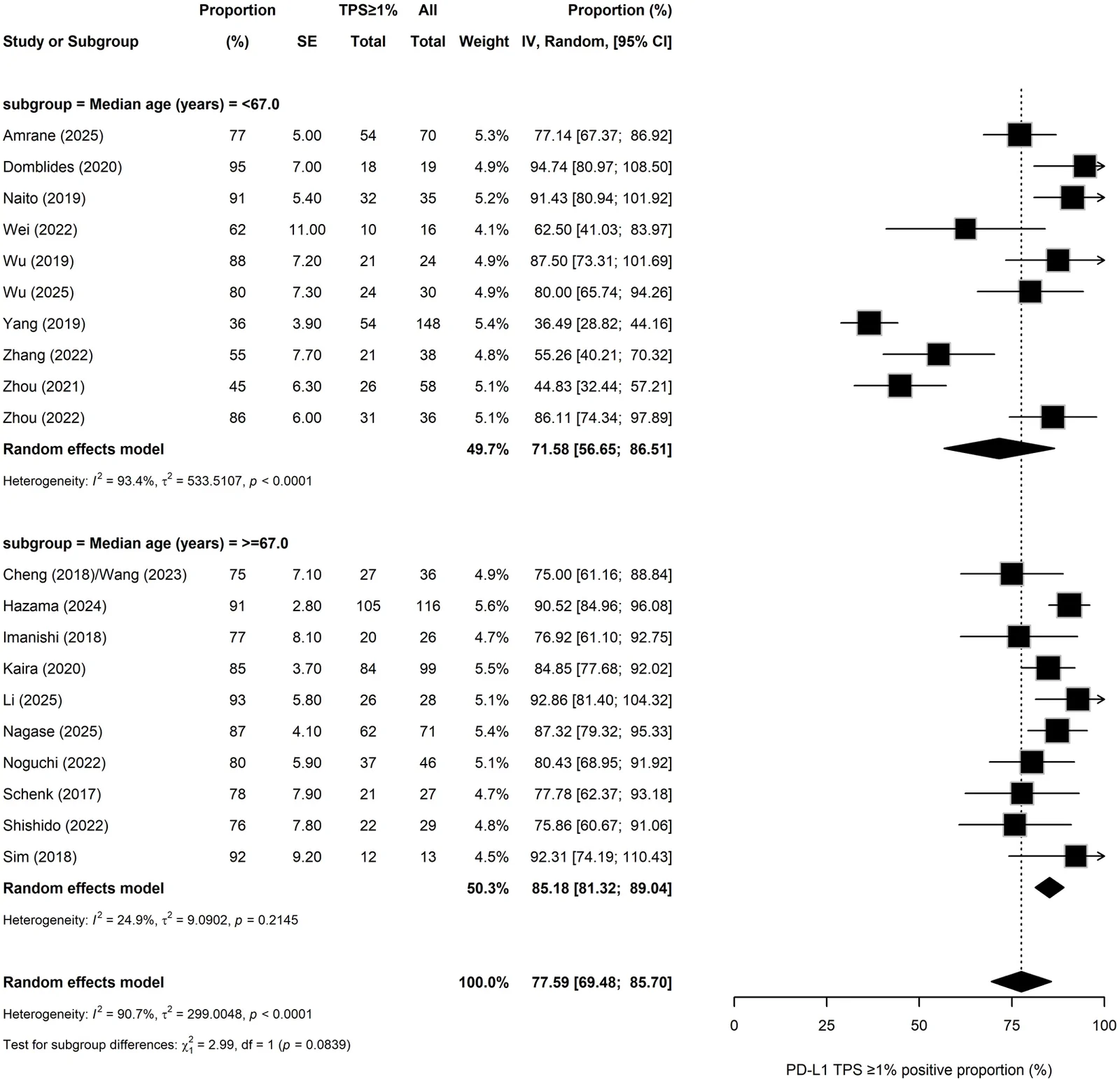


**Supplementary Figure 8:**
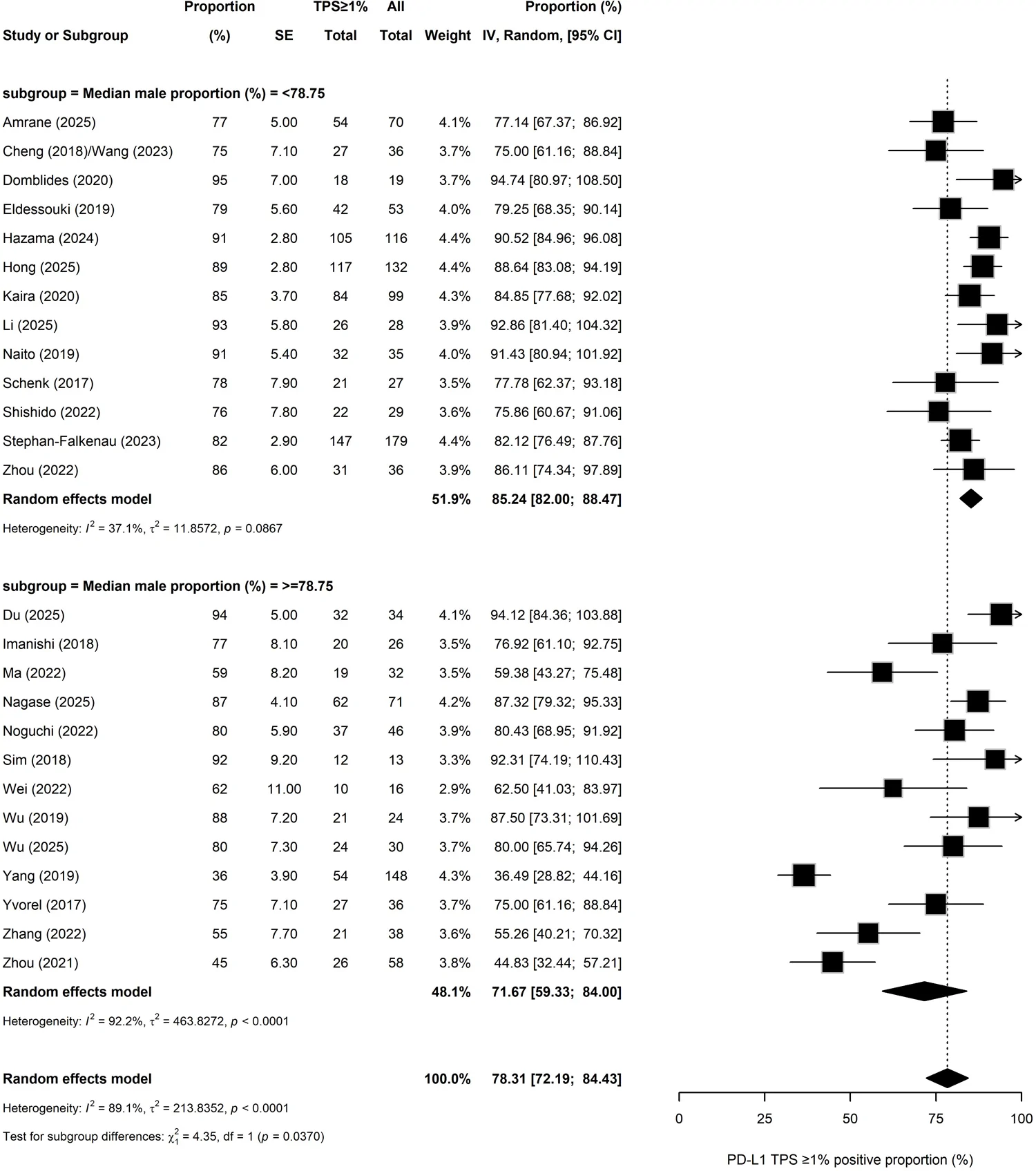


**Supplementary Figure 9:**
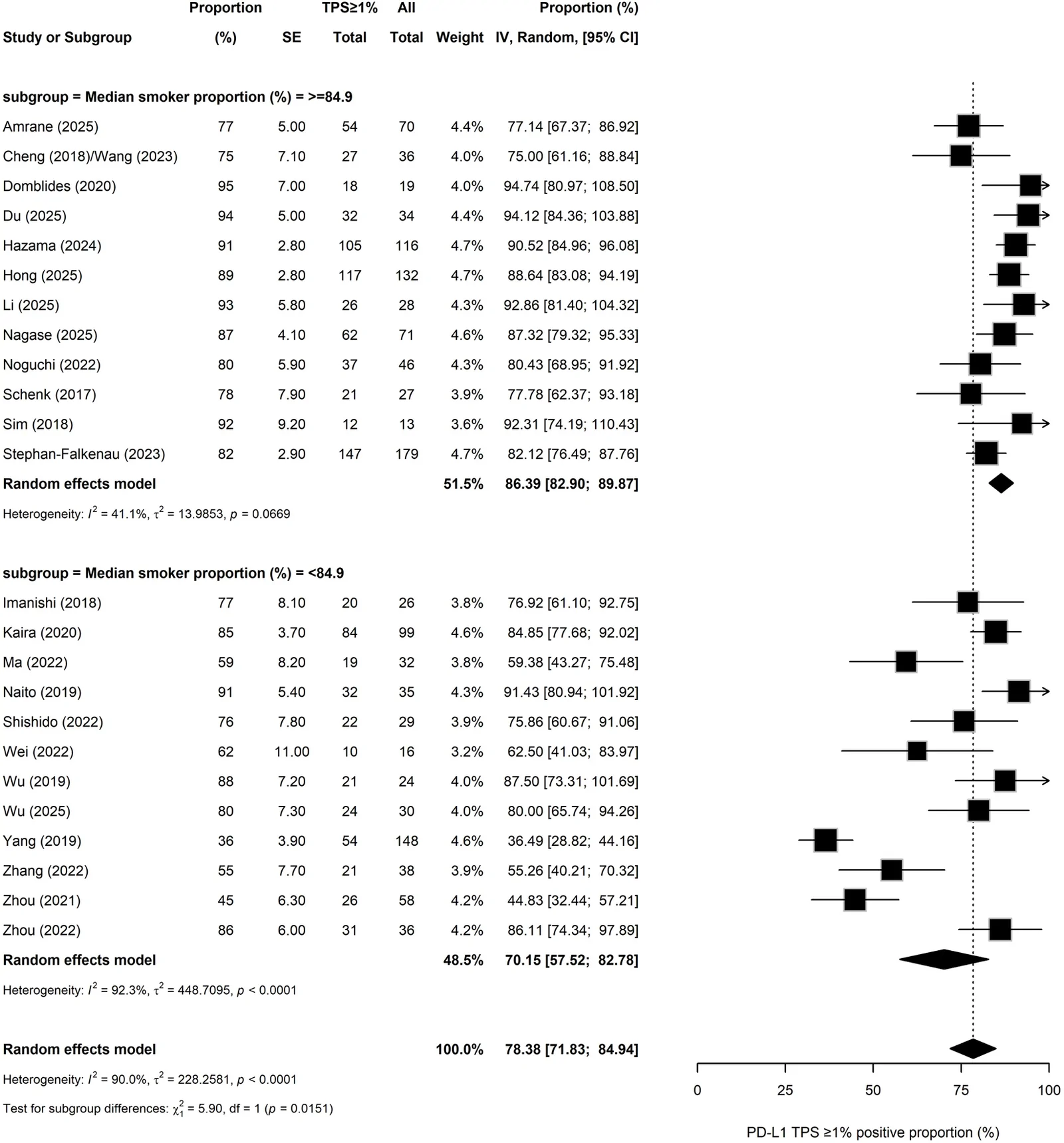


**Supplementary Figure 10:**
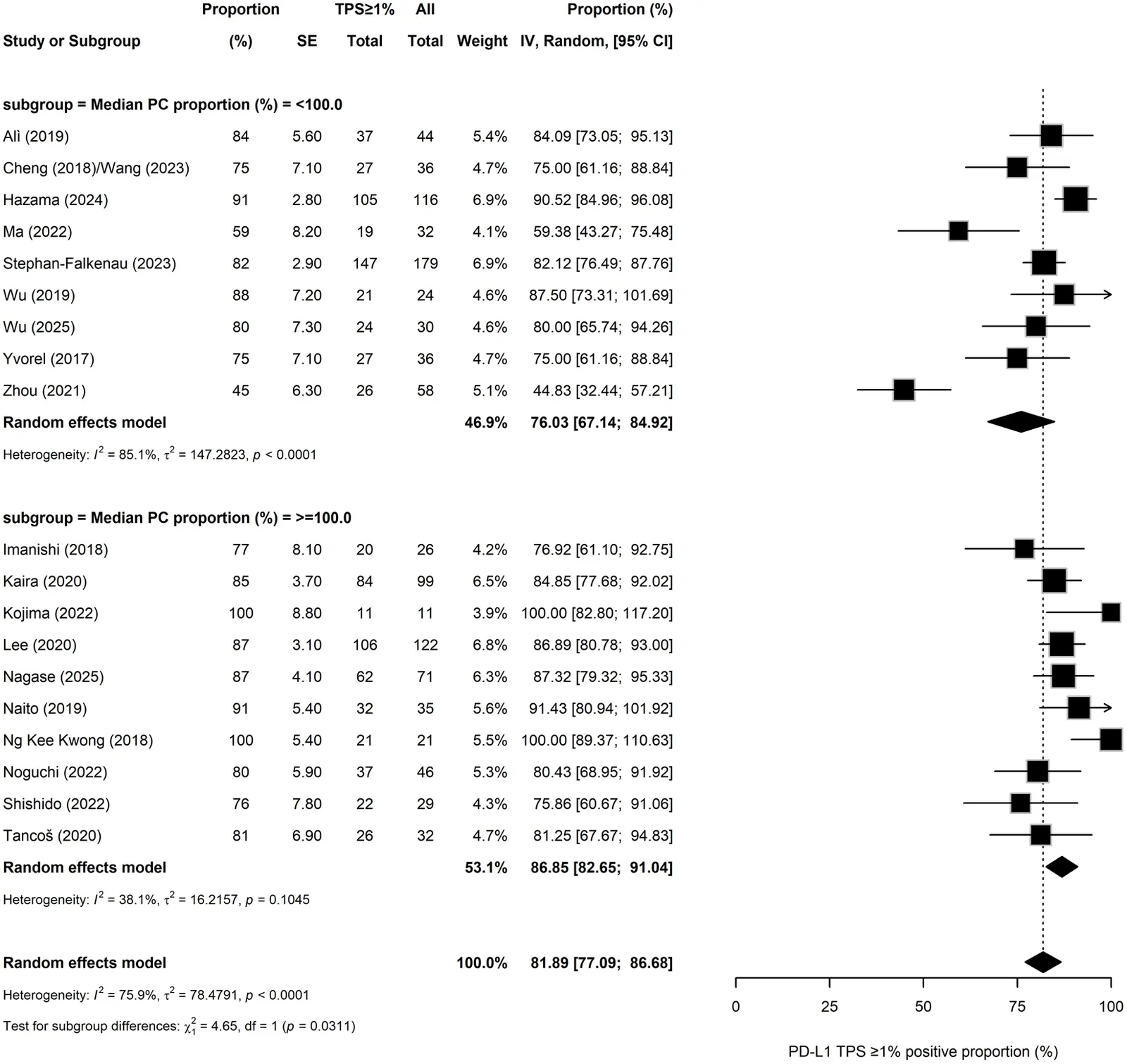


Because the subgroup cutoffs were derived from study-level medians rather than clinically established thresholds, and because the covariates are aggregate study-level characteristics, the subgroup analyses are regarded as exploratory and hypothesis-generating; the complete results are provided in Supplementary Table 3, with forest plots in Supplementary Figures 1–10. For the TPS of 50% or higher threshold, geographic region reported the largest between-subgroup difference, although it did not reach statistical significance (χ^2^ = 3.67, *df* = 1, *p* = 0.055); non-Asian studies reported a numerically higher pooled prevalence (65.59%; 95% CI: 57.35%–73.82%; I^2^ = 78.1%) than Asian studies (52.51%; 95% CI: 41.96%–63.05%; I^2^ = 91.0%). No other covariate reported a significant between-subgroup difference at this threshold (all *p* > 0.05; Supplementary Table 3).

In an exploratory analysis at the 1% threshold, the proportion of ever-smokers reported a statistically significant between-subgroup difference (χ^2^ = 5.90, *df* = 1, *p* = 0.015). Studies with a higher proportion of ever-smokers (≥ 84.9%) had a pooled prevalence of 86.39% (95% CI: 82.90%–89.88%; I^2^ = 41.1%), whereas studies with a lower proportion (< 84.9%) reported a pooled prevalence of 70.15% (95% CI: 57.52%–82.78%; I^2^ = 92.3%). The proportion of pleomorphic carcinoma also reported a significant between-subgroup difference (χ^2^ = 4.65, *df* = 1, *p* = 0.031). Studies with 100% pleomorphic carcinoma had a pooled prevalence of 86.85% (95% CI: 82.65%–91.04%; I^2^ = 38.1%), compared with 76.03% (95% CI: 67.14%–84.92%; I^2^ = 85.1%) in studies with a lower proportion. At this threshold, the proportion of male patients also reported a nominally significant between-subgroup difference (χ^2^ = 4.35, *df* = 1, *p* = 0.037), with a higher pooled prevalence in studies with a lower proportion of male patients (< 78.75%); however, this association was not corroborated in meta-regression (*p* = 0.493) and is therefore regarded as exploratory only. No statistically significant between-subgroup differences were observed for the remaining covariates, including geographic region (all *p* > 0.05; Supplementary Table 3).

### Meta-Regression Analyses

**Figure 3:**
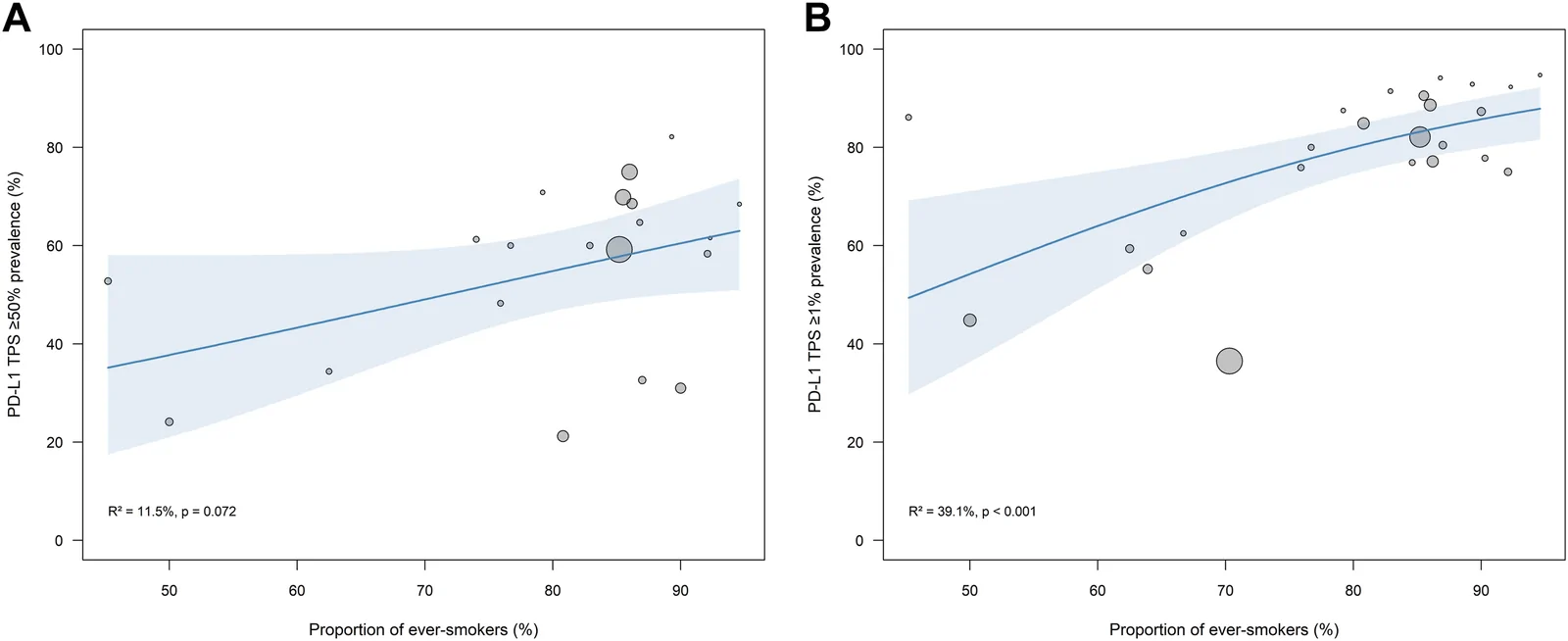
Meta-regression bubble plots of the association between the proportion of ever-smokers and PD-L1 prevalence in pulmonary sarcomatoid carcinoma. (*A*) TPS ≥ 50% threshold (R^2^ = 11.5%, *p* = 0.072); (*B*) TPS ≥ 1% threshold (R^2^ = 39.1%, *p* < 0.001). Each circle represents one study, with circle size proportional to the inverse variance (study weight). The solid line indicates the fitted meta-regression line, and the shaded area represents the 95% confidence interval. PD-L1, programmed death-ligand 1; TPS, tumor proportion score.

**Supplementary Figure 11:**
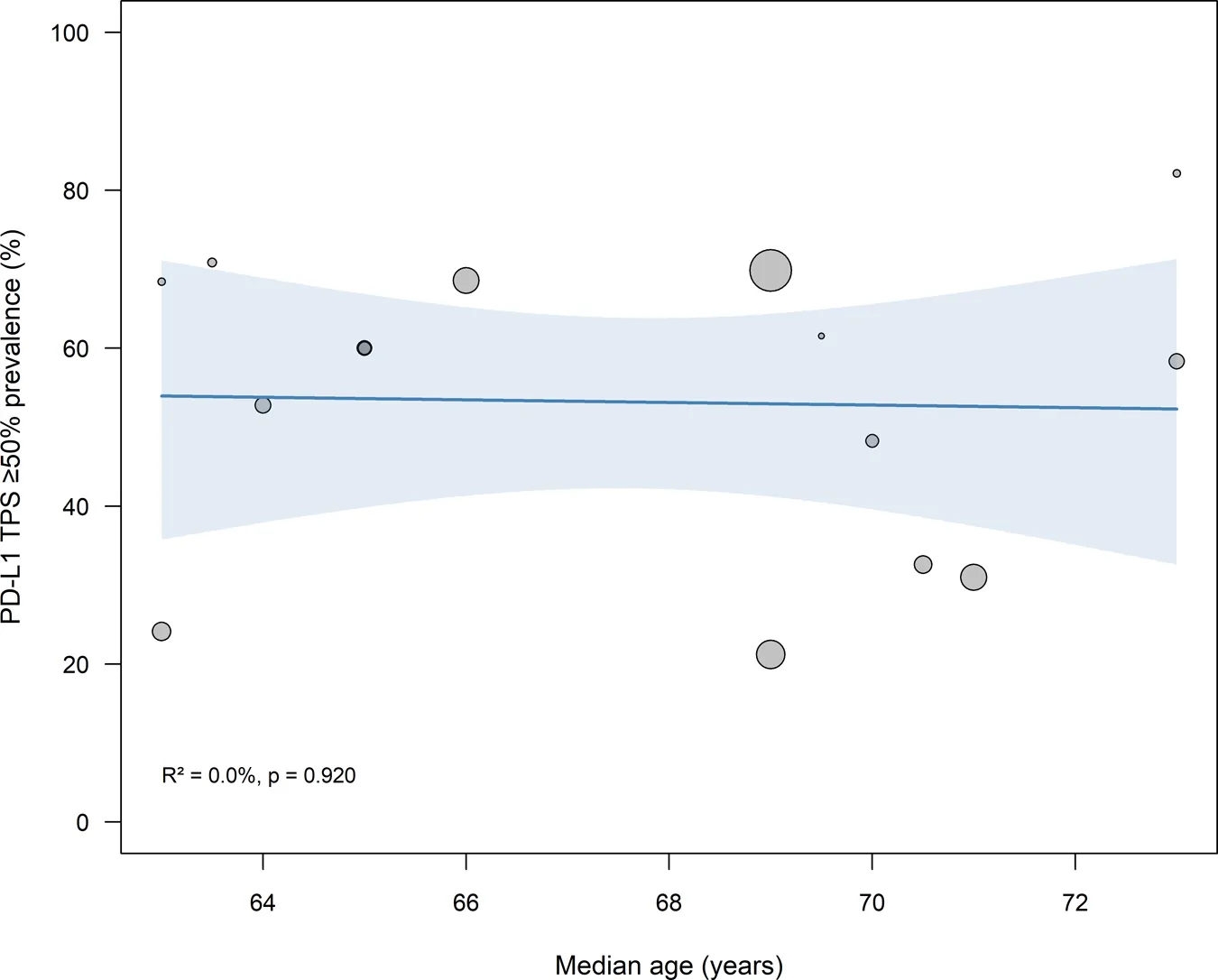


**Supplementary Figure 12:**
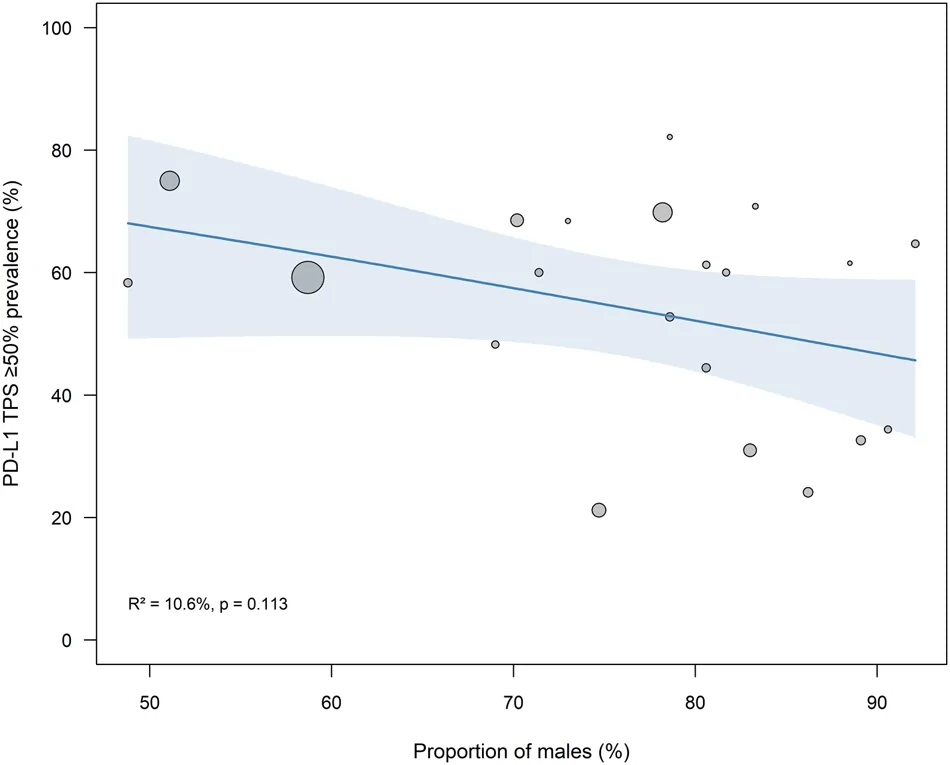


**Supplementary Figure 13:**
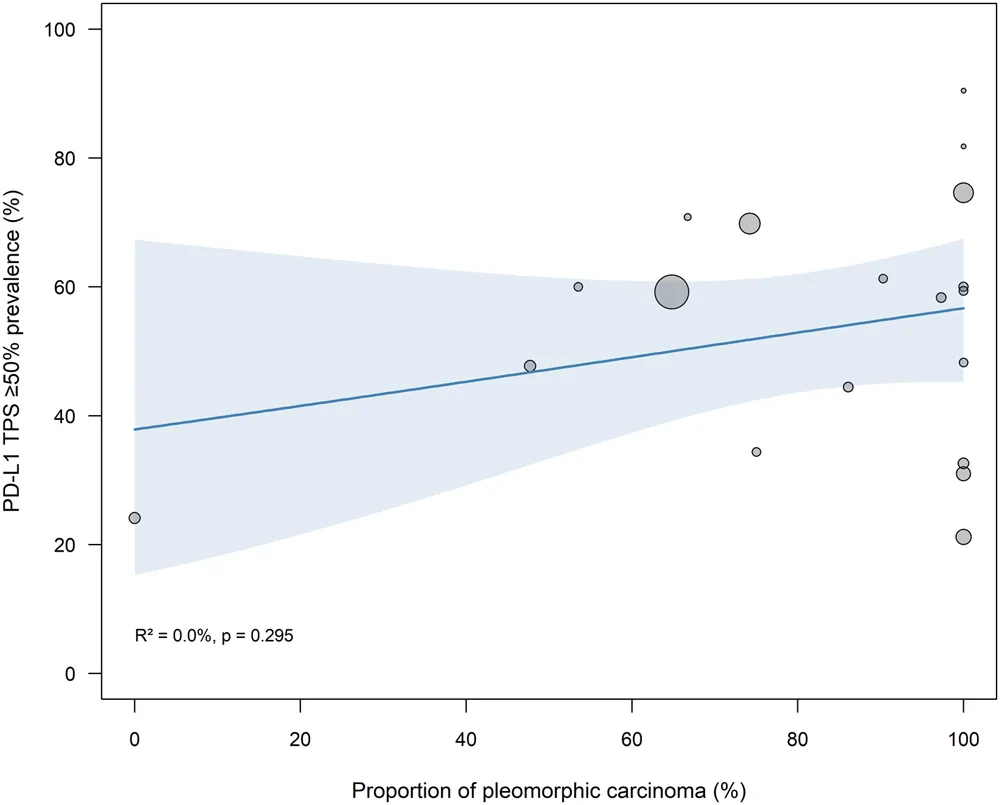


**Supplementary Figure 14:**
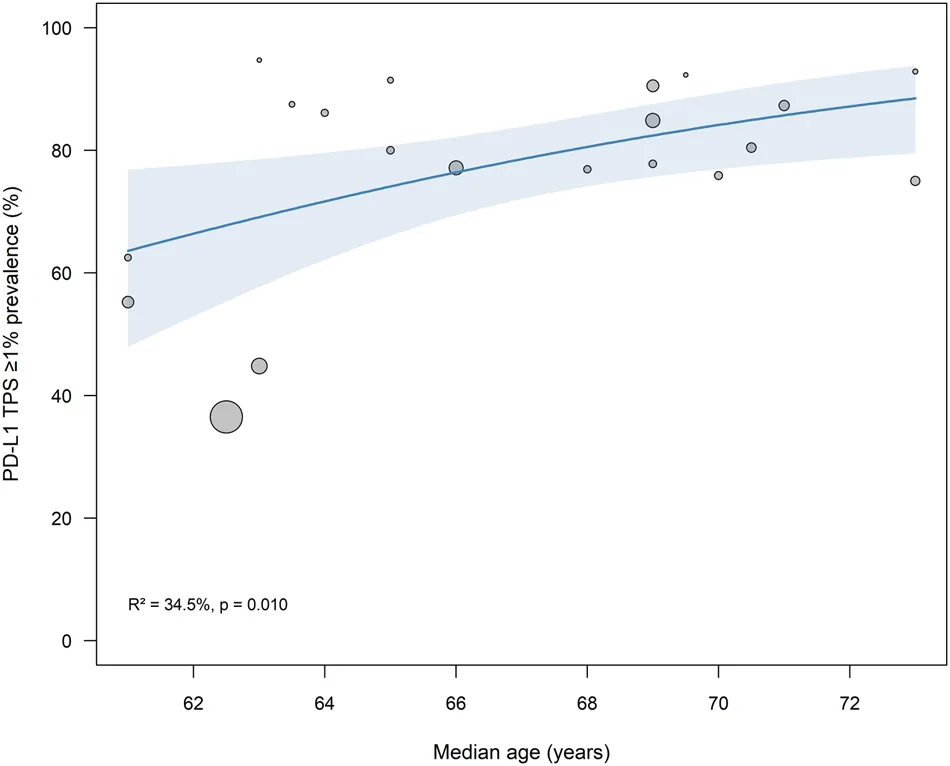


**Supplementary Figure 15:**
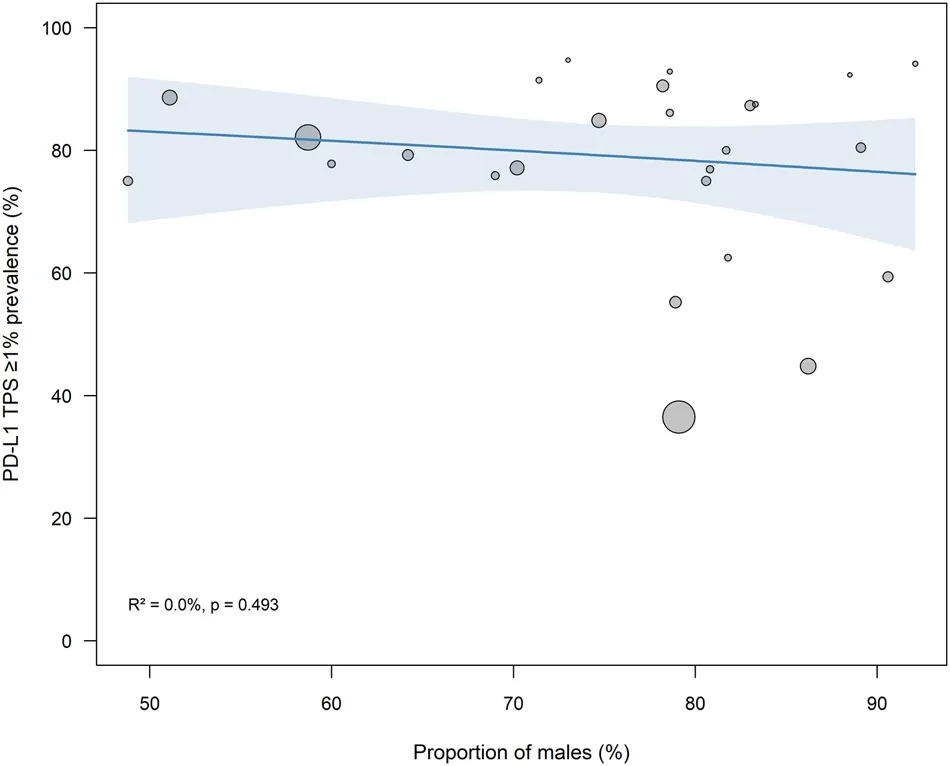


**Supplementary Figure 16:**
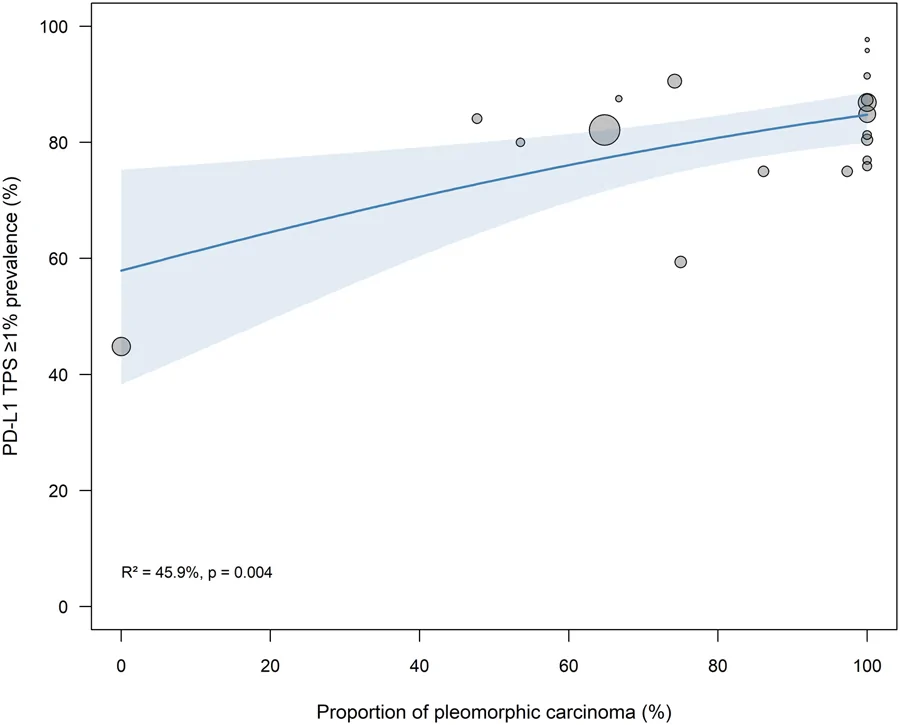


The results of univariate meta-regression analyses are summarized in Table 2, with bubble plots provided in Figure 3 and Supplementary Figures 11–16. For the TPS of 50% or higher threshold, no covariate reported a statistically significant association with PD-L1 prevalence; the geographic region reported a nonsignificant association (R^2^ = 10.0%, *p* = 0.063). The median age, the proportion of men, the proportion of ever-smokers, and the proportion of pleomorphic carcinoma were likewise not significantly associated with PD-L1 prevalence at this threshold (all *p* > 0.05).

**Table 2:** Results of Univariate Meta-Regression Analyses for PD-L1 Expression in Pulmonary Sarcomatoid Carcinoma

Covariate	k	Coefficient (logit)	SE	95% CI	*p*-value	R^2^ (%)	QM (p)
TPS ≥50% threshold							
Region (Non-Asian vs Asian)	27	+0.558	0.300	−0.030 to 1.146	0.063	10.0	3.46 (0.063)
Median age	15	−0.007	0.066	−0.136 to 0.122	0.920	0.0	0.01 (0.920)
Men (%)	21	−0.022	0.014	−0.048 to 0.005	0.113	10.6	2.52 (0.113)
Smoker (%)	20	+0.023	0.013	−0.002 to 0.048	0.072	11.5	3.23 (0.072)
PC (%)	19	+0.008	0.007	−0.007 to 0.022	0.295	0.0	1.10 (0.295)
TPS ≥1% threshold							
Region (Non-Asian vs Asian)	33	+0.223	0.306	−0.377 to 0.824	0.466	0.0	0.53 (0.466)
Median age	20	+0.123	0.048	0.030 to 0.217	0.010∗	34.5	6.69 (0.010)
Men (%)	26	−0.010	0.015	−0.040 to 0.019	0.493	0.0	0.47 (0.493)
Smoker (%)	24	+0.041	0.012	0.017 to 0.064	<0.001∗	39.1	11.43 (<0.001)
PC (%)	19	+0.014	0.005	0.004 to 0.023	0.004∗	45.9	8.38 (0.004)

∗ Statistically significant at *p* < 0.05. Coefficients represent the change in logit-transformed prevalence per unit increase in the covariate. Region is coded as Non-Asian = 1, Asian = 0. Meta-regression was performed using the restricted maximum likelihood method with logit-transformed proportions (metafor package, R version 4.3.3). Abbreviation: k, number of studies; SE, standard error; CI, confidence interval; R^2^, proportion of between-study heterogeneity explained; QM, omnibus test of moderator; PC, pleomorphic carcinoma; TPS, tumor proportion score.

Three covariates reported statistically significant associations for the TPS of 1% or higher threshold. The proportion of pleomorphic carcinoma explained the largest proportion of heterogeneity (R^2^ = 45.9%, *p* = 0.004), followed by the proportion of ever-smokers (R^2^ = 39.1%, *p* < 0.001) and median age (R^2^ = 34.5%, *p* = 0.010). The geographic region and the proportion of men were not significantly associated at this threshold (both *p* > 0.05). The association between the proportion of ever-smokers and PD-L1 prevalence is illustrated in Figure 3, as smoking history is the most readily ascertainable covariate in clinical practice at the time of treatment decision-making.

### Publication Bias

**Supplementary Figure 17:**
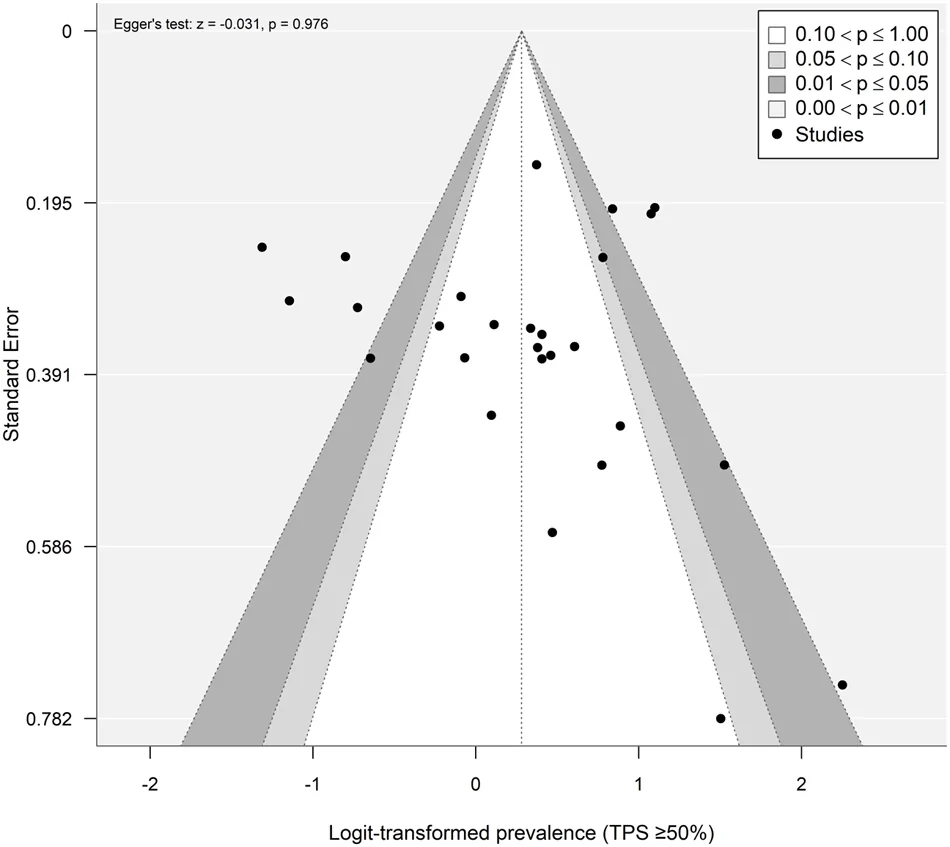


**Supplementary Figure 18:**
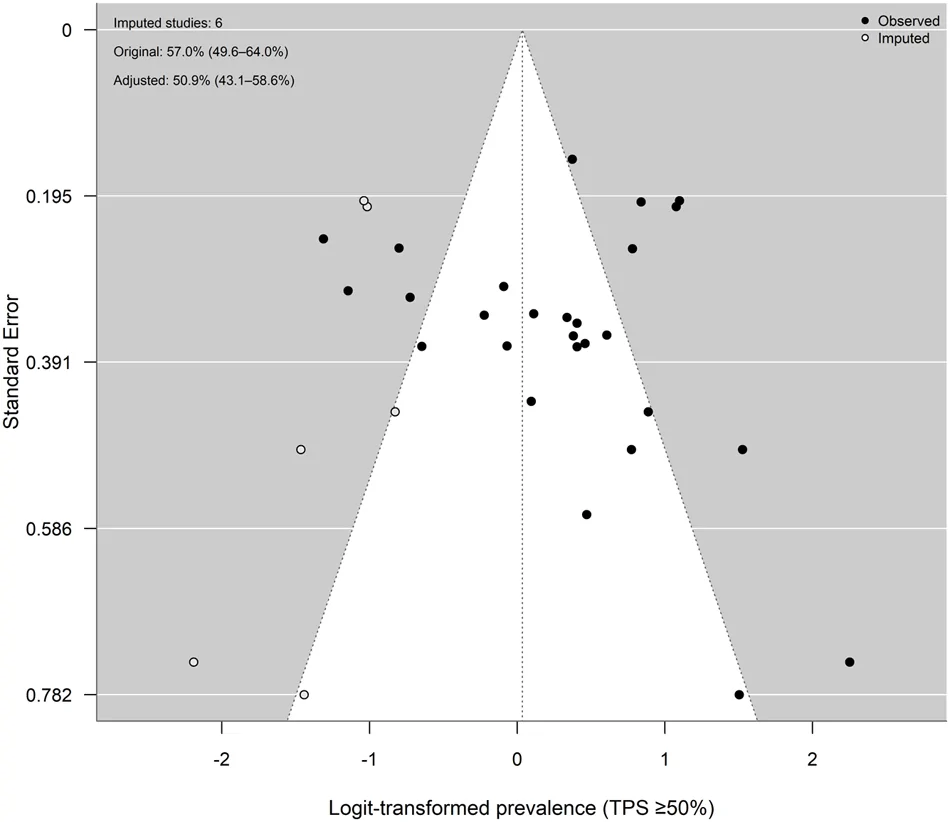


For the TPS of 50% or higher threshold, visual inspection of the contour-enhanced funnel plot reported a relatively symmetrical distribution of studies around the pooled estimate (Supplementary Fig 17). Egger’s regression test confirmed that there was no significant funnel plot asymmetry (*z* = −0.031, *p* = 0.976). Trim-and-fill analysis imputed six studies on the left side of the funnel plot, yielding an adjusted prevalence of 50.9% (95% CI: 43.1%–58.6%), compared with the unadjusted logit-scale estimate of 57.0% (95% CI: 49.6%–64.0%) (Supplementary Fig. 18).

**Supplementary Figure 19:**
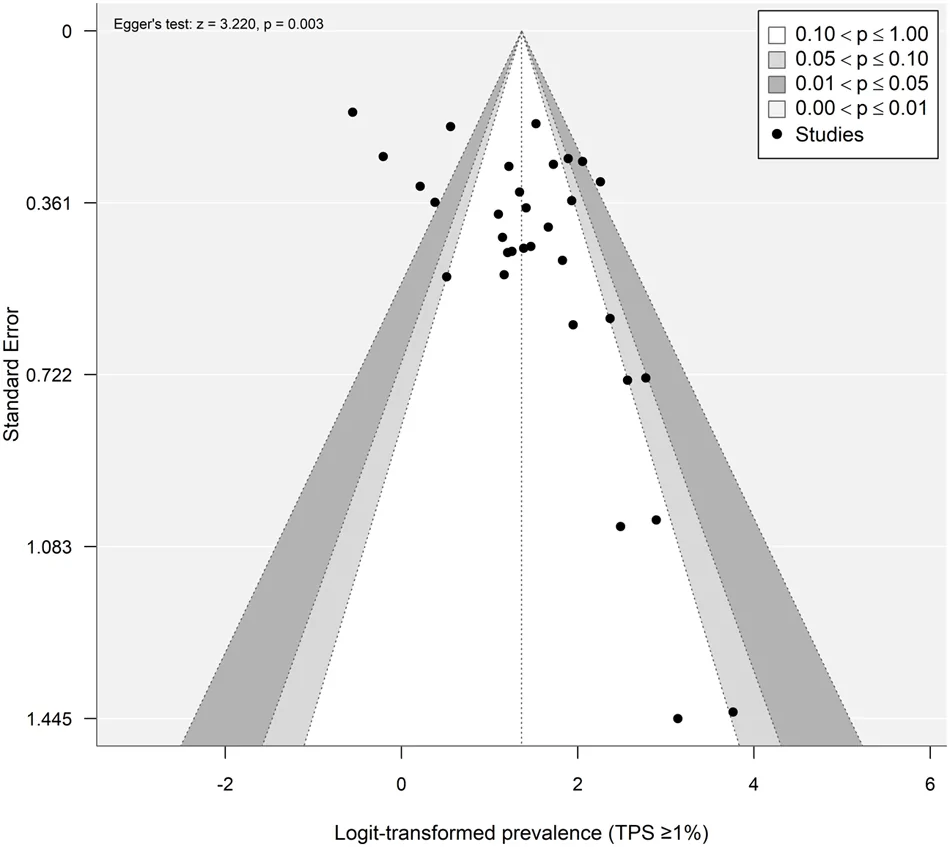


**Supplementary Figure 20:**
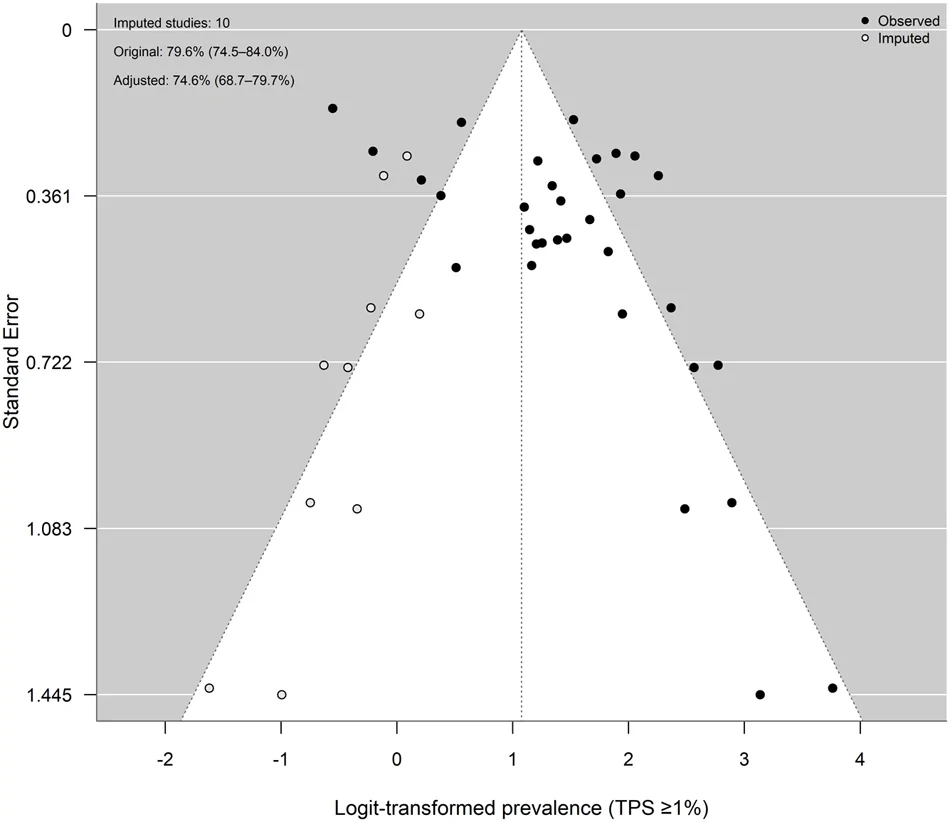


For the TPS of 1% or higher threshold, the contour-enhanced funnel plot found noticeable rightward asymmetry, with several small studies reporting a high PD-L1 prevalence (Supplementary Fig 19). Egger’s regression test confirmed significant funnel plot asymmetry (*z* = 3.220, *p* = 0.003). Trim-and-fill analysis imputed 10 studies on the left side, yielding an adjusted prevalence of 74.6% (95% CI: 68.7%–79.7%), compared with the unadjusted logit-scale estimate of 79.6% (95% CI: 74.5%–84.0%) (Supplementary Fig 20).

### Sensitivity Analysis

**Supplementary Figure 21:**
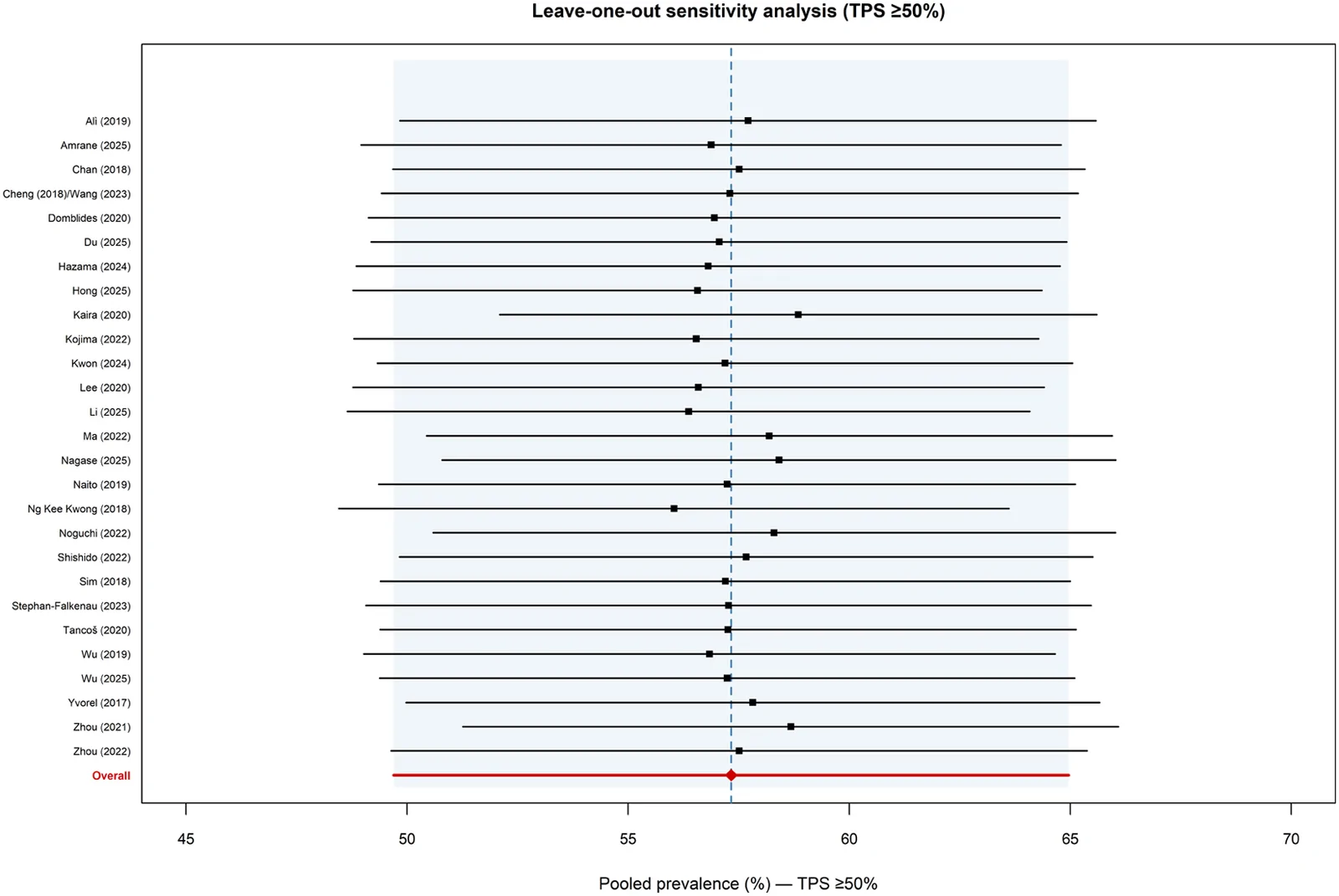


**Supplementary Figure 22:**
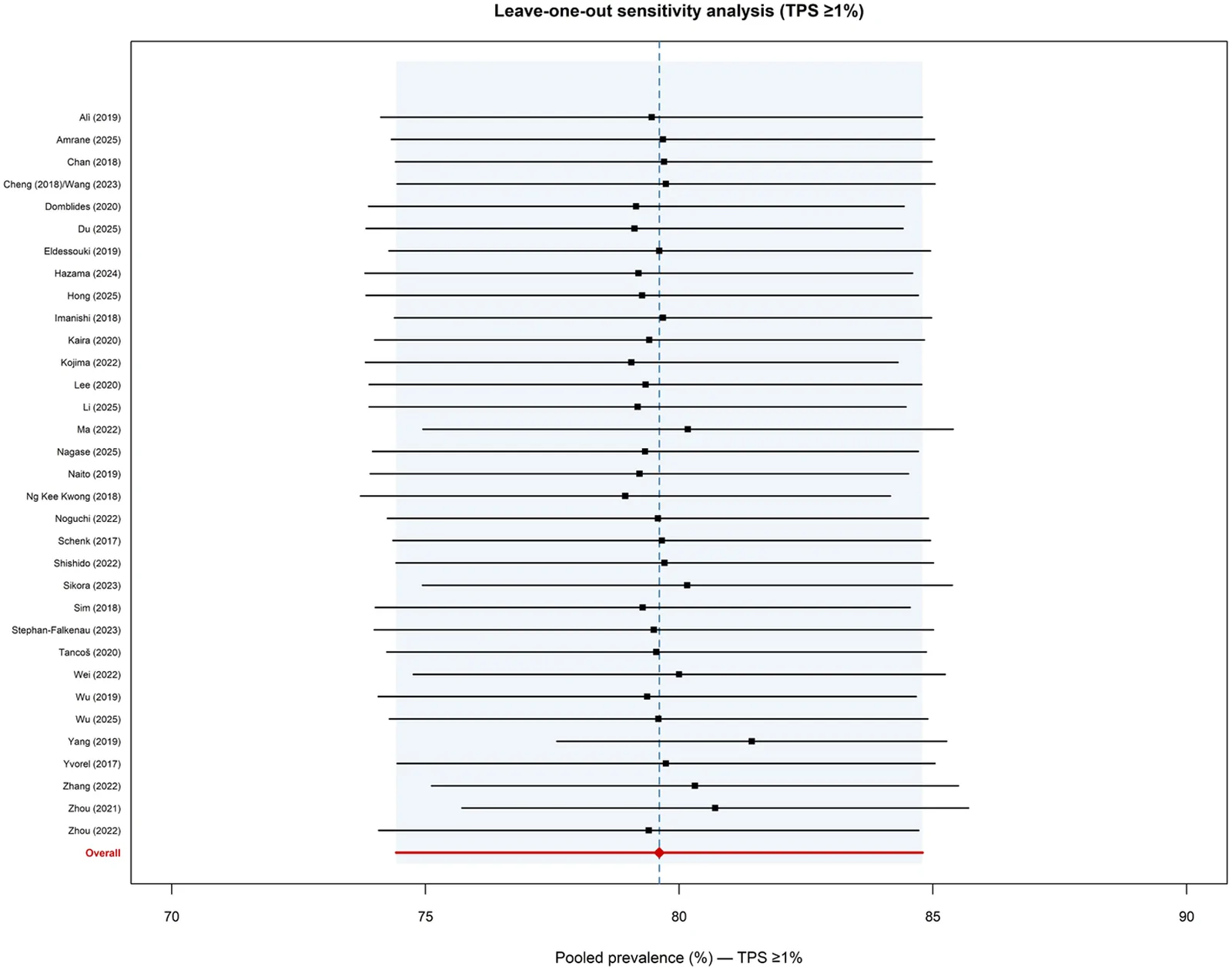


The pooled prevalence estimates in the leave-one-out sensitivity analysis reported minimal variation on the sequential exclusion of each study. For the TPS of 50% or higher threshold, the pooled prevalence ranged from 56.04% to 58.85% across all iterations (overall: 57.33%), and from 78.94% to 81.43% for the TPS of 1% or higher threshold (overall: 79.61%) (Supplementary Fig 21–22).

## Discussion

Although individual studies have reported PD-L1 expression in PSC, no previous systematic review or meta-analysis has quantitatively synthesized these data. To our knowledge, the present study constitutes the first meta-analysis to determine the pooled prevalence of PD-L1 expression in PSC and to identify clinicopathological factors associated with its variability. By synthesizing data from 34 studies encompassing up to 1789 patients, we found that the pooled prevalence of high PD-L1 expression (TPS ≥ 50%) was 57.33% (95% CI: 49.70%–64.96%) and PD-L1 positivity (TPS ≥ 1%) was 79.61% (95% CI: 74.42%–84.79%). These estimates are considerably higher than those reported for conventional NSCLC; the global EXPRESS study reported that only 22% of patients with advanced NSCLC exhibited a TPS of 50% or higher, and 52% exhibited a TPS of 1% or higher.^54^ A Bayesian meta-analysis across 92 studies estimated the prevalence of a TPS of 50% or higher and a TPS of 1% or higher at 27.0% and 58.3%, respectively, in unselected NSCLC.^55^ This higher prevalence—approximately two- to three-fold at the TPS of 50% or higher threshold—underscores the distinctive immunological phenotype of PSC and the potential relevance of immunotherapy in this tumor type.

PD-L1 expression is highly prevalent in PSC and is clinically predictive of ICI efficacy. Lee et al.^10^ reported that patients with high PD-L1 expression achieved an objective response rate of 58.5% with PD-1 and PD-L1 inhibitors, whereas those with low or negative expression reported no objective responses. Babacan et al.^11^ found that PD-L1-positive patients (≥ 1%) attained a median PFS of 14.4 months compared with 2.7 months in PD-L1-negative patients. Given the exceptionally aggressive behavior of PSC and the time required for biomarker testing,^2^^,^^4^^,^^56^ the high population-level PD-L1 prevalence established by the present meta-analysis may help to inform treatment decision-making, particularly in clinical settings where individual PD-L1 results are not yet available.

At the TPS of 50% or higher threshold, non-Asian studies reported a numerically higher pooled prevalence than Asian studies, but this regional difference did not reach statistical significance in either meta-regression (R^2^ = 10.0%, *p* = 0.063) or exploratory subgroup analysis (non-Asian 65.59% versus Asian 52.51%; *p* = 0.055). This numerically higher prevalence in non-Asian studies may nonetheless reflect systematic differences in patient selection and study-level characteristics between populations. Non-Asian studies reported consistently high proportions of ever-smokers (median 89.3%, range 85.2%–94.6%) compared with the wider range observed in Asian studies (median 78.0%, range 45.2%–92.3%). These compositional differences may partially account for the observed disparity. However, at the TPS of 1% or higher threshold, geographic region was not significantly associated with PD-L1 prevalence in meta-regression (*p* = 0.466) or subgroup analysis (*p* = 0.323). Overall, the regional comparison did not reach statistical significance at either threshold and should be interpreted as exploratory rather than as evidence of a robust difference in PD-L1 biology across populations. Other covariates, including the proportion of ever-smokers, median age, proportion of men, and proportion of pleomorphic carcinoma, were not significantly associated with PD-L1 prevalence at the TPS of 50% or higher threshold.

At the TPS of 1% or higher threshold, the proportion of ever-smokers was significantly and positively associated with PD-L1 prevalence in meta-regression (R^2^ = 39.1%, *p* < 0.001). This finding is biologically plausible because smoking-induced mutagenesis elevates tumor mutational burden and consequently upregulates PD-L1 expression as a mechanism of immune evasion.^57^, ^58^, ^59^, ^60^, ^61^, ^62^ Given that PSC is predominantly a disease of smokers, this smoking–PD-L1 axis likely contributes to the high PD-L1 expression observed in this tumor type. Clinically, smoking history is readily ascertainable at the bedside and can serve as a simple indicator to further stratify the expected probability of PD-L1 positivity. Notably, this association was not significant at the TPS of 50% or higher threshold, indicating that the smoking–PD-L1 association in PSC may be more robust for any positivity rather than for high-level expression (Fig 3).

Median age also emerged as a significant predictor of PD-L1 positivity at the TPS of 1% or higher threshold (R^2^ = 34.5%, *p* = 0.010). This may reflect the cumulative accumulation of somatic mutations with age,^63^ although individual-level data were not available, and this association warrants further investigation.

The proportion of pleomorphic carcinoma was significantly associated with PD-L1 positivity at the TPS of 1% or higher threshold, explaining the largest proportion of between-study heterogeneity (R^2^ = 45.9%, *p* = 0.004). Among PSC subtypes, pleomorphic carcinoma uniquely contains both epithelial and sarcomatoid components, reflecting a state of partial epithelial–mesenchymal transition.^64^^,^^65^ The hybrid epithelial-mesenchymal phenotype may maintain both neoantigen presentation capacity and PD-L1 upregulation through adaptive immune resistance mechanisms. However, direct validation in PSC subtype-comparative studies is lacking, which warrants further investigation.

Regarding publication bias, the Egger’s test reported no evidence of funnel plot asymmetry for a TPS of 50% or higher (*p* = 0.976), whereas significant asymmetry was detected for a TPS of 1% or higher (*p* = 0.003). However, the observed asymmetry likely reflects heterogeneity in study-level characteristics rather than selective nonpublication. Trim-and-fill adjusted prevalence estimates (50.9% for TPS ≥ 50% and 74.6% for TPS ≥ 1%) remained substantially high, consistent with the overall conclusion that PD-L1 expression is highly prevalent in PSC.

This study has several limitations. First, all included studies were observational, and the inherent heterogeneity in study design, patient selection, and PD-L1 scoring practices may have contributed to the substantial between-study heterogeneity (I^2^ > 85% for both thresholds). Nevertheless, leave-one-out sensitivity analysis found stable pooled estimates regardless of the excluded study. Although all included studies used commercially available antibody clones, many did not specify the particular clone, precluding clone-specific analyses. The Blueprint PD-L1 immunohistochemistry (IHC) Assay Comparison Project^66^ reported comparable analytical performance among the major assays, although SP142 yielded lower tumor cell staining, which may have introduced some measurement variability. Second, meta-regression was limited to study-level covariates, and the ecological fallacy precludes direct inference to individual patients. Third, multivariate meta-regression was not performed because of the limited number of studies relative to covariates, and potential confounding between covariates could not be excluded. Fourth, this review was restricted to English-language publications, which may have introduced selection bias.

## Conclusion

To our knowledge, this study is the first meta-analysis to quantify PD-L1 expression and its prevalence in PSC. PD-L1 expression is remarkably prevalent in this rare malignancy, with approximately 57% of patients exhibiting high expression (TPS ≥ 50%) and 80% exhibiting any PD-L1 positivity (TPS ≥ 1%). At the TPS of 1% or higher threshold, the proportion of ever-smokers, the proportion of pleomorphic carcinoma, and median age were significant predictors of PD-L1 expression, whereas no covariate was a significant predictor at the TPS of 50% or higher threshold. Given the aggressive clinical course of PSC and the limited efficacy of conventional chemotherapy, these findings provide quantitative evidence regarding PD-L1 positivity that may inform treatment decision-making for advanced PSC, particularly in clinical settings where individual biomarker results are pending. Future studies incorporating individual patient data, standardized antibody clones, and multivariate analyses are warranted to further elucidate the determinants of PD-L1 expression in PSC and to validate the clinical utility of population-level prevalence estimates for guiding immunotherapy decisions.

## CRediT Authorship Contribution Statement

**Yusuke Hamakawa**: Conceptualization, Methodology, Formal analysis, Data curation, Writing – original draft, Writing – review & editing.

**Aya Shiba**: Investigation, Data curation, Writing – review & editing.

**Yohei Noguchi:** Investigation, Writing – review & editing.

**Hiroyuki Shinohara**: Investigation, Writing – review & editing.

**Yuko Higashi**: Investigation, Writing – review & editing.

**Masaharu Aga**: Investigation, Writing – review & editing.

**Kazuhito Miyazaki**: Investigation, Writing – review & editing.

**Yuri Taniguchi**: Investigation, Writing – review & editing.

**Yuki Misumi**: Investigation, Writing – review & editing.

**Yoko Agemi**: Investigation, Writing – review & editing.

**Tsuneo Shimokawa**: Investigation, Writing – review & editing.

**Hiroaki Okamoto**: Supervision, Writing – review & editing.

**Nobuyuki Horita**: Methodology, Formal analysis, Writing – review & editing.

**Yukiko Nakamura**: Conceptualization, Supervision, Writing – review & editing.

## Data statement

The data that support the findings of this study are available from the corresponding author on reasonable request.

## Disclosure

The authors declare that they have no known competing financial interests or personal relationships that could have appeared to influence the work reported in this article.

## Supplementary Data

Supplementary Figures Legend

Supplementary References
