# Supplementary material for: Prevalence of High Programmed Death-Ligand 1 Expression in Pulmonary Sarcomatoid Carcinoma: A Systematic Review and Meta-Analysis
Source: JTO Clin Res Rep. 2026 Jul 10;7(10):101046. doi: 10.1016/j.jtocrr.2026.101046 (PMC13571916; doi:10.1016/j.jtocrr.2026.101046)
Supplement: Supplementary file 1 [file mmc1.docx]

**Supplementary Appendix.** Studies reporting PD-L1 in pulmonary sarcomatoid carcinoma that were excluded at full-text review because the antibody and/or scoring method were not interchangeable with 22C3-based tumour proportion score (TPS).

| **Study** | **PD-L1 antibody clone** | **Scoring method** | **Positivity cut-off** | **Reason for exclusion** |
| --- | --- | --- | --- | --- |
| Velcheti 2013 | 5H1 (non-commercial; provided by L. Chen, Yale) | Automated quantitative immunofluorescence (QIF/AQUA); continuous AQUA score measured within the cytokeratin-defined tumour mask | None (continuous score, arbitrary units) | PD-L1 measured as a continuous immunofluorescence score with a non-commercial antibody and no membrane-based threshold; cannot be converted to a 22C3-equivalent TPS ≥1% or ≥50%. |
| Kim 2015 | E1L3N | Immunohistochemistry; combined intensity- and proportion-based membranous/cytoplasmic tumour-cell scoring | ≥10% tumour cells | Combined intensity- and proportion-based membranous/cytoplasmic scoring with a ≥10% threshold is not interchangeable with 22C3 membrane-only TPS at the ≥1% or ≥50% cut-off. |
| Vieira 2016 | 5H1 (non-commercial; provided by L. Chen, Yale) | Immunohistochemistry; membranous tumour-cell staining (EnVision/DAB detection) | ≥5% tumour-cell membrane staining | Non-commercial antibody (clone 5H1, reported not to correlate with the E1L3N assay) scored at a non-standard ≥5% threshold; TPS ≥1%/≥50% strata not reported. |
| Chang 2016 | Proteintech (non-companion-diagnostic antibody) | Immunohistochemistry; moderate-to-strong membranous tumour-cell staining | ≥5% tumour cells | Antibody outside the validated companion-diagnostic clones and a ≥5%-only threshold; TPS ≥1%/≥50% strata not reported. |
| Ağaçkıran 2021 | SP263 | Immunohistochemistry; combined membranous/cytoplasmic tumour-cell staining | ≥25% | Combined membranous/cytoplasmic staining scored at a ≥25% threshold is not interchangeable with 22C3 membrane-only TPS at the ≥1% or ≥50% cut-off. |

TPS, tumour proportion score (percentage of viable tumour cells with partial or complete membrane staining at any intensity), as defined for the 22C3 pharmDx assay. The validated companion-diagnostic clones referenced for comparability are 22C3, 28-8, SP263 and SP142.
