# Supplementary material for: Prevalence of High Programmed Death-Ligand 1 Expression in Pulmonary Sarcomatoid Carcinoma: A Systematic Review and Meta-Analysis
Source: JTO Clin Res Rep. 2026 Jul 10;7(10):101046. doi: 10.1016/j.jtocrr.2026.101046 (PMC13571916; doi:10.1016/j.jtocrr.2026.101046)
Supplement: Supplementary file 2 [file mmc2.docx]

**Supplementary Figure Legends**

**Supplementary Figure 1.** Forest plot of subgroup analysis stratified by geographic region for the TPS ≥ 50% threshold.

TPS, tumor proportion score

**Supplementary Figure 2.** Forest plot of subgroup analysis stratified by median age for the TPS ≥ 50% threshold.

TPS, tumor proportion score

**Supplementary Figure 3.** Forest plot of subgroup analysis stratified by the proportion of male patients for the TPS ≥ 50% threshold.

TPS, tumor proportion score

**Supplementary Figure 4.** Forest plot of subgroup analysis stratified by the proportion of ever-smokers for the TPS ≥ 50% threshold.

TPS, tumor proportion score

**Supplementary Figure 5.** Forest plot of subgroup analysis stratified by the proportion of pleomorphic carcinoma for the TPS ≥ 50% threshold.

TPS, tumor proportion score

**Supplementary Figure 6.** Forest plot of subgroup analysis stratified by geographic region for the TPS ≥ 1% threshold.

TPS, tumor proportion score

**Supplementary Figure 7.** Forest plot of subgroup analysis stratified by median age for the TPS ≥ 1% threshold.

TPS, tumor proportion score

**Supplementary Figure 8.** Forest plot of subgroup analysis stratified by the proportion of male patients for the TPS ≥ 1% threshold.

TPS, tumor proportion score

**Supplementary Figure 9.** Forest plot of subgroup analysis stratified by the proportion of ever-smokers for the TPS ≥ 1% threshold.

TPS, tumor proportion score

**Supplementary Figure 10.** Forest plot of subgroup analysis stratified by the proportion of pleomorphic carcinoma for the TPS ≥ 1% threshold.

TPS, tumor proportion score

**Supplementary Figure 11.** Meta-regression bubble plot of the association between median age and PD-L1 prevalence at the TPS ≥ 50% threshold. Each circle represents one study, with circle size proportional to the inverse variance (study weight). The solid line indicates the fitted meta-regression line, and the shaded area represents the 95% confidence interval.

PD-L1, programmed death-ligand 1; TPS, tumor proportion score

**Supplementary Figure 12.** Meta-regression bubble plot of the association between the proportion of male patients and PD-L1 prevalence at the TPS ≥ 50% threshold. Each circle represents one study, with circle size proportional to the inverse variance (study weight). The solid line indicates the fitted meta-regression line, and the shaded area represents the 95% confidence interval.

PD-L1, programmed death-ligand 1; TPS, tumor proportion score

**Supplementary Figure 13.** Meta-regression bubble plot of the association between the proportion of pleomorphic carcinoma and PD-L1 prevalence at the TPS ≥ 50% threshold. Each circle represents one study, with circle size proportional to the inverse variance (study weight). The solid line indicates the fitted meta-regression line, and the shaded area represents the 95% confidence interval.

PD-L1, programmed death-ligand 1; TPS, tumor proportion score

**Supplementary Figure 14.** Meta-regression bubble plot of the association between median age and PD-L1 prevalence at the TPS ≥ 1% threshold. Each circle represents one study, with circle size proportional to the inverse variance (study weight). The solid line indicates the fitted meta-regression line, and the shaded area represents the 95% confidence interval.

PD-L1, programmed death-ligand 1; TPS, tumor proportion score

**Supplementary Figure 15.** Meta-regression bubble plot of the association between the proportion of male patients and PD-L1 prevalence at the TPS ≥ 1% threshold. Each circle represents one study, with circle size proportional to the inverse variance (study weight). The solid line indicates the fitted meta-regression line, and the shaded area represents the 95% confidence interval.

PD-L1, programmed death-ligand 1; TPS, tumor proportion score

**Supplementary Figure 16.** Meta-regression bubble plot of the association between the proportion of pleomorphic carcinoma and PD-L1 prevalence at the TPS ≥ 1% threshold. Each circle represents one study, with circle size proportional to the inverse variance (study weight). The solid line indicates the fitted meta-regression line, and the shaded area represents the 95% confidence interval.

PD-L1, programmed death-ligand 1; TPS, tumor proportion score

**Supplementary Figure 17.** Contour-enhanced funnel plot for the TPS ≥ 50% threshold. The shaded regions correspond to significance levels of p < 0.10, p < 0.05, and p < 0.01. Egger's regression test result is displayed in the upper left corner.

TPS, tumor proportion score

**Supplementary Figure 18.** Trim-and-fill funnel plot for the TPS ≥ 50% threshold. Filled circles represent observed studies, and open circles represent imputed studies. The number of imputed studies and the original and adjusted pooled prevalence estimates are displayed.

TPS, tumor proportion score

**Supplementary Figure 19.** Contour-enhanced funnel plot for the TPS ≥ 1% threshold. The shaded regions correspond to significance levels of p < 0.10, p < 0.05, and p < 0.01. Egger's regression test result is displayed in the upper left corner.

TPS, tumor proportion score

**Supplementary Figure 20.** Trim-and-fill funnel plot for the TPS ≥ 1% threshold. Filled circles represent observed studies, and open circles represent imputed studies. The number of imputed studies and the original and adjusted pooled prevalence estimates are displayed.

TPS, tumor proportion score

**Supplementary Figure 21.** Leave-one-out sensitivity analysis for the TPS ≥ 50% threshold. Each row represents the pooled prevalence estimate after excluding the corresponding study. The blue dashed line and shaded band indicate the overall pooled estimate and its 95% confidence interval. The red diamond at the bottom represents the overall estimate from the full model.

TPS, tumor proportion score

**Supplementary Figure 22.** Leave-one-out sensitivity analysis for the TPS ≥ 1% threshold. Each row represents the pooled prevalence estimate after excluding the corresponding study. The blue dashed line and shaded band indicate the overall pooled estimate and its 95% confidence interval. The red diamond at the bottom represents the overall estimate from the full model.

TPS, tumor proportion score
