# Supplementary material for: Prevalence of High Programmed Death-Ligand 1 Expression in Pulmonary Sarcomatoid Carcinoma: A Systematic Review and Meta-Analysis
Source: JTO Clin Res Rep. 2026 Jul 10;7(10):101046. doi: 10.1016/j.jtocrr.2026.101046 (PMC13571916; doi:10.1016/j.jtocrr.2026.101046)
Supplement: Supplementary file 3 [file mmc3.docx]

**Supplementary References: Full Citations for the Included Studies**

Full bibliographic citations for each of the 34 studies included in the quantitative synthesis and in the methodological quality assessment (Supplementary Table 2). Each citation is keyed to the first-author/year label used in Table 1 and Supplementary Table 2. One entry (Cheng 2018 / Wang 2023) combines two reports of the same cohort, both of which are cited. Conference abstracts are indicated.

**1. [Alì (2019)]** Alì G, Bruno R, Poma AM, et al. Whole transcriptome targeted gene quantification provides new insights on pulmonary sarcomatoid carcinomas. *Sci Rep*. 2019;9(1):3536. doi: [10.1038/s41598-019-40016-8](https://doi.org/10.1038/s41598-019-40016-8).

**2. [Amrane (2025)]** Amrane K, Cabarrou B, Justeau G, et al. Efficacy of first-line treatments for advanced pulmonary sarcomatoid carcinomas: a real-world analysis. *ESMO Open*. 2025;10(8):105343. doi: [10.1016/j.esmoop.2025.105343](https://doi.org/10.1016/j.esmoop.2025.105343).

**3. [Chan (2018)]** Chan AWH, Tong JHM, Kwan JSH, et al. Assessment of programmed cell death ligand-1 expression by 4 diagnostic assays and its clinicopathological correlation in a large cohort of surgical resected non-small cell lung carcinoma. *Mod Pathol*. 2018;31(9):1381-1390. doi: [10.1038/s41379-018-0053-3](https://doi.org/10.1038/s41379-018-0053-3).

**4a. [Cheng (2018) / Wang (2023)]** Cheng H, Sharma J, Su C, et al. Differential expression of immune checkpoints (PD-L1, HHLA2, B7x and B7H3) and their association with driver mutations in pulmonary sarcomatoid carcinoma (PSC). *Ann Oncol*. 2018;29(Suppl 8):viii672. *[conference abstract]*

**4b. [Cheng (2018) / Wang (2023)]** Wang F, Cali Daylan AE, Deng L, et al. Heterogeneous expression of PD-L1, B7x, B7-H3, and HHLA2 in pulmonary sarcomatoid carcinoma and the related regulatory signaling pathways. *Cancers (Basel)*. 2023;15(13).  [doi: 10.3390/cancers15133372](https://doi.org/10.3390/cancers15133372)

**5. [Domblides (2020)]** Domblides C, Leroy K, Monnet I, et al. Efficacy of immune checkpoint inhibitors in lung sarcomatoid carcinoma. *J Thorac Oncol*. 2020;15(5):860-866. doi: [10.1016/j.jtho.2020.01.014](https://doi.org/10.1016/j.jtho.2020.01.014).

**6. [Du (2025)]** Du H, Song X, Wu F. Evaluation of the effectiveness and safety profile of first-line immune checkpoint inhibitors combined with chemotherapy in pulmonary sarcomatoid carcinoma. *Lung Cancer (Auckl)*. 2025;16:73-83. doi: [10.2147/LCTT.S494990](https://doi.org/10.2147/LCTT.S494990).

**7. [Eldessouki (2019)]** Eldessouki I, Hassan R, Ma PC, Karim NA. Predictive value of K-Ras subset mutations and PD-L1 in pulmonary sarcomatoid carcinoma (PSC). *J Thorac Oncol*. 2019;14(10):S384. *[conference abstract]*

**8. [Hazama (2024)]** Hazama D, Nakahama K, Kodama H, et al. Effectiveness and safety of immune checkpoint inhibitors alone or in combination with chemotherapy in pulmonary sarcomatoid carcinoma. *JTO Clin Res Rep*. 2024;5(1):100613. doi: [10.1016/j.jtocrr.2023.100613](https://doi.org/10.1016/j.jtocrr.2023.100613).

**9. [Hong (2025)]** Hong L, Di Federico A, Liu B, et al. Distinct clinicogenomic features and immunotherapy associations in pulmonary sarcomatoid carcinoma: a multicenter retrospective study. *J Thorac Oncol*. 2025. doi: [10.1016/j.jtho.2025.07.121](https://doi.org/10.1016/j.jtho.2025.07.121).

**10. [Imanishi (2018)]** Imanishi N, Hirai A, Yoneda K, et al. Programmed death-ligand 1 (PD-L1) expression in pleomorphic carcinoma of the lung. *J Surg Oncol*. 2018;117(7):1563-1569. doi: [10.1002/jso.25041](https://doi.org/10.1002/jso.25041).

**11. [Kaira (2020)]** Kaira K, Shimizu K, Endoh H, et al. Prognostic significance of tumor immunity in surgically resected pulmonary pleomorphic carcinoma. *Anticancer Res*. 2020;40(1):261-269. doi: [10.21873/anticanres.13948](https://doi.org/10.21873/anticanres.13948).

**12. [Kojima (2022)]** Kojima K, Sakamoto T, Kasai T, Atagi S, Yoon H. A quantitative evaluation of the histological type dependence of the programmed death-ligand 1 expression in non-small cell lung cancer including various adenocarcinoma subtypes: a cross-sectional study. *Jpn J Clin Oncol*. 2022;52(3):281-285. doi: [10.1093/jjco/hyab202](https://doi.org/10.1093/jjco/hyab202).

**13. [Kwon (2024)]** Kwon HJ, Lee S, Han YB, et al. Genomic landscape of pulmonary sarcomatoid carcinoma. *Cancer Res Treat*. 2024;56(2):442-454. doi: [10.4143/crt.2023.764](https://doi.org/10.4143/crt.2023.764).

**14. [Lee (2020)]** Lee J, La Choi Y, Jung HA, et al. Outstanding clinical efficacy of PD-1/PD-L1 inhibitors for pulmonary pleomorphic carcinoma. *Eur J Cancer*. 2020;132:150-158. doi: [10.1016/j.ejca.2020.03.029](https://doi.org/10.1016/j.ejca.2020.03.029).

**15. [Li (2025)]** Li JJ, Saab J, Bray V, et al. Histopathological associations, molecular findings and clinical outcomes of patients with non-small cell lung carcinoma with MET alterations: a 3-year retrospective Australian case series. *Pathology*. 2026;58:41-51. doi: [10.1016/j.pathol.2025.07.009](https://doi.org/10.1016/j.pathol.2025.07.009).

**16. [Ma (2022)]** Ma Y, Li WS, Li ZZ, et al. Immunophenotyping of pulmonary sarcomatoid carcinoma. *Front Immunol*. 2022;13. doi: [10.3389/fimmu.2022.976739](https://doi.org/10.3389/fimmu.2022.976739).

**17. [Nagase (2025)]** Nagase W, Kudo Y, Matsubayashi J, et al. Immunotherapy-extended survival in patients with recurrent pulmonary pleomorphic carcinoma following surgery. *Eur J Surg Oncol*. 2025;51(3):109565. doi: [10.1016/j.ejso.2024.109565](https://doi.org/10.1016/j.ejso.2024.109565).

**18. [Naito (2019)]** Naito M, Tamiya A, Takeda M, et al. A high PD-L1 expression in pulmonary pleomorphic carcinoma correlates with parietal-pleural invasion and might predict a poor prognosis. *Intern Med*. 2019;58(7):921-927. doi: [10.2169/internalmedicine.1462-18](https://doi.org/10.2169/internalmedicine.1462-18).

**19. [Ng Kee Kwong (2018)]** Ng Kee Kwong F, Laggner U, McKinney O, Croud J, Rice A, Nicholson AG. Expression of PD-L1 correlates with pleomorphic morphology and histological patterns of non-small-cell lung carcinomas. *Histopathology*. 2018;72(6):1024-1032. doi: [10.1111/his.13466](https://doi.org/10.1111/his.13466).

**20. [Noguchi (2022)]** Noguchi M, Menju T, Nakajima N, et al. High programmed death ligand 1 expression in carcinomatous components predicts a poor prognosis in pulmonary pleomorphic carcinoma. *Surg Today*. 2022;52(7):1090-1095. doi: [10.1007/s00595-021-02439-7](https://doi.org/10.1007/s00595-021-02439-7).

**21. [Schenk (2017)]** Schenk E, Boland J, Mansfield A, Aubry MC, Dietz A. Local and systemic immunity predict survival in patients with pulmonary sarcomatoid carcinoma. *Med Oncol*. 2017;34(8):140. doi: [10.1007/s12032-017-1000-8](https://doi.org/10.1007/s12032-017-1000-8).

**22. [Shishido (2022)]** Shishido Y, Aoyama A, Hara S, et al. Ringed fluorodeoxyglucose uptake predicted poor prognosis after resection of pulmonary pleomorphic carcinoma. *J Cardiothorac Surg*. 2022;17(1):47. doi: [10.1186/s13019-022-01799-6](https://doi.org/10.1186/s13019-022-01799-6).

**23. [Sikora (2023)]** Sikora K, Langfort R. PD-L1 expression in surgically resected lung pleomorphic carcinoma - one-centre study of 107 patients. *J Thorac Oncol*. 2023;18(11):S243-S244. *[conference abstract]*

**24. [Sim (2018)]** Sim JK, Chung SM, Choi JH, et al. Clinical and molecular characteristics of pulmonary sarcomatoid carcinoma. *Korean J Intern Med*. 2018;33(4):737-744. doi: [10.3904/kjim.2017.245](https://doi.org/10.3904/kjim.2017.245).

**25. [Stephan-Falkenau (2023)]** Stephan-Falkenau S, Streubel A, Mairinger T, et al. Integrated clinical, molecular and immunological characterization of pulmonary sarcomatoid carcinomas reveals an immune escape mechanism that may influence therapeutic strategies. *Int J Mol Sci*. 2023;24(13). doi: [10.3390/ijms241310558](https://doi.org/10.3390/ijms241310558).

**26. [Tancoš (2020)]** Tancoš V, Farkašová A, Kviatkovská Z, et al. Non-small cell lung carcinomas with a minor sarcomatoid component and pleomorphic carcinomas are associated with high expression of programmed death ligand 1. *Pathol Res Pract*. 2020;216(12):153238. doi: [10.1016/j.prp.2020.153238](https://doi.org/10.1016/j.prp.2020.153238).

**27. [Wei (2022)]** Wei JW, Hao Y, Xiang J, et al. The prognostic impact of immune checkpoint inhibitors for the treatment of pulmonary sarcomatoid carcinoma: a multicenter retrospective study. *Neoplasma*. 2022;69(6):1437-1444. doi: [10.4149/neo_2022_220617N644](https://doi.org/10.4149/neo_2022_220617N644).

**28. [Wu (2019)]** Wu XD, Huang Y, Li Y, Wang Q, Wang HQ, Jiang L. 18F-FDG PET/CT imaging in pulmonary sarcomatoid carcinoma and correlation with clinical and genetic findings. *Ann Nucl Med*. 2019;33(9):647-656. doi: [10.1007/s12149-019-01374-5](https://doi.org/10.1007/s12149-019-01374-5).

**29. [Wu (2025)]** Wu MY, Zhou N, Guan M, Wang YY, Wang YZ. Efficacy of immune checkpoint inhibitors in pulmonary sarcomatoid carcinoma and predictive potential of mutated TP53. *Lung Cancer*. 2025;199:108068. doi: [10.1016/j.lungcan.2024.108068](https://doi.org/10.1016/j.lungcan.2024.108068).

**30. [Yang (2019)]** Yang Z, Xu J, Li R, Gao Y, He J. PD-L1 and CD47 co-expression in pulmonary sarcomatoid carcinoma: a predictor of poor prognosis and potential targets of future combined immunotherapy. *J Cancer Res Clin Oncol*. 2019;145(12):3055-3065. doi: [10.1007/s00432-019-03023-w](https://doi.org/10.1007/s00432-019-03023-w).

**31. [Yvorel (2017)]** Yvorel V, Patoir A, Casteillo F, et al. PD-L1 expression in pleomorphic, spindle cell and giant cell carcinoma of the lung is related to TTF-1, p40 expression and might indicate a worse prognosis. *PLoS One*. 2017;12(7):e0180346. doi: [10.1371/journal.pone.0180346](https://doi.org/10.1371/journal.pone.0180346).

**32. [Zhang (2022)]** Zhang CY, Li ZX, Zhang YX, et al. Genomic variations and immune-related features of TMB, PD-L1 expression and CD8+ T cell infiltration in Chinese pulmonary sarcomatoid carcinoma. *Int J Gen Med*. 2022;15:4209-4220. doi: [10.2147/IJGM.S357659](https://doi.org/10.2147/IJGM.S357659).

**33. [Zhou (2021)]** Zhou F, Huang Y, Cai W, et al. The genomic and immunologic profiles of pure pulmonary sarcomatoid carcinoma in Chinese patients. *Lung Cancer*. 2021;153:66-72. doi: [10.1016/j.lungcan.2021.01.006](https://doi.org/10.1016/j.lungcan.2021.01.006).

**34. [Zhou (2022)]** Zhou F, Guo HY, Zhou XL, et al. Immune checkpoint inhibitors plus chemotherapy in patients with locally advanced or metastatic pulmonary sarcomatoid carcinoma: a multicentric real-world study. *Ther Adv Med Oncol*. 2022;14:17588359221136759. doi: [10.1177/17588359221136759](https://doi.org/10.1177/17588359221136759).
