# Supplementary material for: Prevalence of High Programmed Death-Ligand 1 Expression in Pulmonary Sarcomatoid Carcinoma: A Systematic Review and Meta-Analysis
Source: JTO Clin Res Rep. 2026 Jul 10;7(10):101046. doi: 10.1016/j.jtocrr.2026.101046 (PMC13571916; doi:10.1016/j.jtocrr.2026.101046)
Supplement: Supplementary file 4 [file mmc4.docx]

**Supplementary Table 1.** Literature search strategies for each database.

| **Database** | **Records** | **Search strategy** |
| --- | --- | --- |
| PubMed | 373 | (pulmonary OR lung OR respiratory) AND (sarcomatoid OR pleomorphic OR spindle-cell OR giant-cell OR carcinosarcoma OR blastoma) AND (PD-L1 OR PD-1 OR PDL1 OR PD1 OR "programmed cell death") |
| Web of Science | 281 | TS=((pulmonary OR lung OR respiratory) AND (sarcomatoid OR pleomorphic OR spindle-cell OR giant-cell OR carcinosarcoma OR blastoma) AND (PD-L1 OR PD-1 OR PDL1 OR PD1 OR "programmed cell death")) |
| Cochrane CENTRAL | 17 | Title/Abstract/Keyword: (pulmonary OR lung OR respiratory) AND (sarcomatoid OR pleomorphic OR spindle-cell OR giant-cell OR carcinosarcoma OR blastoma) AND (PD-L1 OR PD-1 OR PDL1 OR PD1 OR "programmed cell death") |
| **Total** | **671** |  |

*All databases were searched from inception through 19 November 2025. No date restrictions were applied.*

*After removing 183 duplicates, 488 unique records were screened based on titles and abstracts.*
