# Supplementary material for: Prevalence of High Programmed Death-Ligand 1 Expression in Pulmonary Sarcomatoid Carcinoma: A Systematic Review and Meta-Analysis
Source: JTO Clin Res Rep. 2026 Jul 10;7(10):101046. doi: 10.1016/j.jtocrr.2026.101046 (PMC13571916; doi:10.1016/j.jtocrr.2026.101046)
Supplement: Supplementary file 5 [file mmc5.docx]

**Supplementary Table 2.** Methodological quality assessment of included studies using the JBI Critical Appraisal Checklist for Studies Reporting Prevalence Data

| **Study** | **Q1** | **Q2** | **Q3** | **Q4** | **Q5** | **Q6** | **Q7** | **Q8** | **Q9** | **Score** |
| --- | --- | --- | --- | --- | --- | --- | --- | --- | --- | --- |
| Alì (2019) | Y | Y | Y | Y | Y | Y | Y | Y | Y | **9/9** |
| Amrane (2025) | Y | Y | Y | Y | Y | Y | Y | Y | Y | **9/9** |
| Chan (2018) | Y | Y | U | Y | Y | Y | Y | Y | Y | **8/9** |
| Cheng (2018) / Wang (2023) | Y | Y | Y | Y | Y | Y | Y | Y | Y | **9/9** |
| Domblides (2020) | Y | Y | U | Y | Y | Y | Y | Y | Y | **8/9** |
| Du (2025) | Y | Y | Y | Y | Y | Y | Y | Y | Y | **9/9** |
| Eldessouki (2019) | Y | U | Y | N | U | Y | U | Y | U | **4/9** |
| Hazama (2024) | Y | Y | Y | Y | Y | Y | Y | Y | Y | **9/9** |
| Hong (2025) | Y | Y | Y | Y | Y | Y | Y | Y | Y | **9/9** |
| Imanishi (2018) | Y | Y | U | Y | Y | Y | Y | Y | Y | **8/9** |
| Kaira (2020) | Y | Y | Y | Y | Y | Y | U | Y | Y | **8/9** |
| Kojima (2022) | Y | Y | U | Y | Y | Y | Y | Y | Y | **8/9** |
| Kwon (2024) | Y | Y | Y | Y | Y | Y | Y | Y | Y | **9/9** |
| Lee (2020) | Y | Y | Y | Y | Y | Y | Y | Y | Y | **9/9** |
| Li (2025) | Y | Y | U | Y | Y | Y | Y | Y | Y | **8/9** |
| Ma (2022) | Y | Y | Y | Y | Y | Y | Y | Y | Y | **9/9** |
| Nagase (2025) | Y | Y | U | Y | Y | Y | Y | Y | Y | **8/9** |
| Naito (2019) | Y | Y | Y | Y | Y | Y | Y | Y | Y | **9/9** |
| Ng Kee Kwong (2018) | Y | Y | U | Y | Y | Y | Y | Y | Y | **8/9** |
| Noguchi (2022) | Y | Y | Y | Y | Y | Y | Y | Y | Y | **9/9** |
| Schenk (2017) | Y | Y | U | Y | Y | Y | Y | Y | Y | **8/9** |
| Shishido (2022) | Y | Y | U | Y | Y | Y | Y | Y | Y | **8/9** |
| Sikora (2023) | Y | U | Y | N | U | Y | U | Y | U | **4/9** |
| Sim (2018) | Y | Y | U | Y | Y | Y | Y | Y | Y | **8/9** |
| Stephan-Falkenau (2023) | Y | Y | Y | Y | Y | Y | Y | Y | Y | **9/9** |
| Tancoš (2020) | Y | Y | Y | Y | Y | Y | Y | Y | Y | **9/9** |
| Wei (2022) | Y | Y | Y | Y | N | Y | Y | Y | N | **7/9** |
| Wu (2019) | Y | Y | Y | Y | Y | U | Y | Y | Y | **8/9** |
| Wu (2025) | Y | Y | Y | Y | N | Y | Y | Y | N | **7/9** |
| Yang (2019) | Y | Y | Y | Y | Y | Y | Y | Y | Y | **9/9** |
| Yvorel (2017) | Y | Y | Y | Y | Y | Y | Y | Y | Y | **9/9** |
| Zhang (2022) | Y | Y | Y | Y | Y | Y | Y | Y | Y | **9/9** |
| Zhou (2021) | Y | Y | Y | Y | Y | Y | Y | Y | Y | **9/9** |
| Zhou (2022) | N | Y | Y | Y | Y | Y | Y | Y | Y | **8/9** |

Y, Yes; N, No; U, Unclear.

**Q1,** Was the sample frame appropriate to address the target population? **Q2,** Were study participants sampled in an appropriate way? **Q3,** Was the sample size adequate? **Q4,** Were the study subjects and the setting described in detail? **Q5,** Was the data analysis conducted with sufficient coverage of the identified sample? **Q6,** Were valid methods used for the identification of the condition? **Q7,** Was the condition measured in a standard, reliable way for all participants? **Q8,** Was there appropriate statistical analysis? **Q9,** Was the response rate adequate, and if not, was the low response rate managed appropriately?
