# Supplementary material for: Prevalence of High Programmed Death-Ligand 1 Expression in Pulmonary Sarcomatoid Carcinoma: A Systematic Review and Meta-Analysis
Source: JTO Clin Res Rep. 2026 Jul 10;7(10):101046. doi: 10.1016/j.jtocrr.2026.101046 (PMC13571916; doi:10.1016/j.jtocrr.2026.101046)
Supplement: Supplementary file 6 [file mmc6.docx]

**Supplementary Table 3.** Summary of exploratory subgroup analyses for PD-L1 prevalence in pulmonary sarcomatoid carcinoma.

| **Subgroup variable** | **Group** | **k** | **Pooled prevalence, % (95% CI)** | **I² (%)** | **χ² (df)** | **p-value** |
| --- | --- | --- | --- | --- | --- | --- |
| **TPS ≥50% threshold** | | | | | | |
| Region | Non-Asian | 10 | 65.59 (57.35–73.82) | 78.1 | 3.67 (1) | 0.055 |
|  | Asian | 17 | 52.51 (41.96–63.05) | 91.0 |  |  |
| Median age | <69.0 years | 7 | 57.34 (42.71–71.97) | 86.0 | 0.39 (1) | 0.533 |
|  | ≥69.0 years | 8 | 50.21 (33.28–67.15) | 93.6 |  |  |
| Male (%) | <78.6% | 9 | 58.69 (45.60–71.78) | 93.0 | 0.76 (1) | 0.384 |
|  | ≥78.6% | 12 | 51.05 (39.90–62.20) | 85.5 |  |  |
| Smoker (%) | ≥85.35% | 10 | 61.15 (49.87–72.43) | 88.4 | 2.16 (1) | 0.142 |
|  | <85.35% | 10 | 48.68 (36.45–60.91) | 89.4 |  |  |
| PC (%) | <97.3% | 9 | 52.39 (41.37–63.42) | 86.2 | 0.09 (1) | 0.770 |
|  | ≥97.3% | 10 | 55.30 (39.22–71.37) | 94.0 |  |  |
| **TPS ≥1% threshold** | | | | | | |
| Region | Non-Asian | 13 | 82.50 (77.22–87.78) | 71.7 | 0.98 (1) | 0.323 |
|  | Asian | 20 | 77.60 (69.45–85.75) | 91.4 |  |  |
| Median age | <67.0 years | 10 | 71.58 (56.65–86.51) | 93.4 | 2.99 (1) | 0.084 |
|  | ≥67.0 years | 10 | 85.18 (81.32–89.04) | 24.9 |  |  |
| Male (%) | <78.75% | 13 | 85.24 (82.00–88.47) | 37.1 | 4.35 (1) | 0.037* |
|  | ≥78.75% | 13 | 71.67 (59.33–84.00) | 92.2 |  |  |
| Smoker (%) | ≥84.9% | 12 | 86.39 (82.90–89.88) | 41.1 | 5.90 (1) | 0.015* |
|  | <84.9% | 12 | 70.15 (57.52–82.78) | 92.3 |  |  |
| PC (%) | 100% | 10 | 86.85 (82.65–91.04) | 38.1 | 4.65 (1) | 0.031* |
|  | <100% | 9 | 76.03 (67.14–84.92) | 85.1 |  |  |

*k, number of studies; CI, confidence interval; PC, pleomorphic carcinoma.*

*These subgroup analyses are exploratory and hypothesis-generating. Subgroup cutoffs were determined by the median value of each covariate among the studies included in the respective analysis and do not represent clinically established thresholds. Pooled prevalences were estimated using random-effects (DerSimonian–Laird) models, and between-subgroup differences were assessed using a chi-squared test (tau² estimated separately within each subgroup).*

** Statistically significant at p < 0.05.*
